# Loss of Beclin 1 primes colorectal cancer cells for Immunogenic necroptosis via transcriptional de-repression of RIPK1/RIPK3/MLKL axis

**DOI:** 10.1007/s11033-025-11037-6

**Published:** 2025-09-19

**Authors:** Hatice Esenkaya, Filiz Ucan Turkmen

**Affiliations:** 1 Division of Biomolecular and Cellular Medicine, Department of Laboratory Medicine, Karolinska Institutet, Huddinge, Sweden; 2 Life Sciences, Molecular Biology and Genetics, Kilis, Turkey

**Keywords:** Beclin 1, Necroptosis, Colorectal cancer, Apoptosis resistance, RIPK1/RIPK3/MLKL axis

## Abstract

**Background:**

Colorectal cancer (CRC) frequently develops resistance to apoptotic cues, creating a clinical imperative to explore alternative forms of programmed cell death. Necroptosis, a regulated form of necrosis driven by RIPK1, RIPK3, and MLKL, is increasingly recognised for its immunogenic potential, yet the transcriptional control of this pathway remains poorly understood. Here, we identify Beclin 1, a canonical autophagy regulator, as a key transcriptional suppressor of necroptotic programming.

**Methods and results:**

Using siRNA-mediated knockdown (KD) in HT-29 CRC cells, we observed a significant reduction in Beclin 1 protein levels, accompanied by a 2.4–2.9-fold upregulation of RIPK1, RIPK3, and MLKL transcripts. Western blot analysis revealed modest increases in phosphorylated RIPK1(pRIPK1) and pMLKL, indicating functional sensitisation to necroptotic death without full pathway execution.

**Conclusions:**

These findings Suggest that Beclin 1 maintains cellular Survival not solely through autophagy but also by repressing necroptosis at the transcriptional level. Its loss reconfigures CRC cell fate, predisposing them to inflammatory, caspase-independent death. Targeting Beclin 1 may therefore expose a previously unrecognised vulnerability in apoptosis-resistant CRC, opening new avenues for necroptosis-based immunotherapeutic interventions.

**Supplementary Information:**

The online version contains supplementary material available at 10.1007/s11033-025-11037-6.

## Introduction

Colorectal cancer (CRC) remains one of the most prevalent malignancies worldwide and a major contributor to cancer-related mortality [1]. A central feature of its pathogenesis is the ability of tumour cells to evade apoptotic death, often through mutations in the TP53 gene, overexpression of anti-apoptotic proteins such as BCL-2 [2], or caspase inactivation [3]. This apoptotic resistance impairs not only therapeutic efficacy but also immune clearance, allowing malignant cells to persist and evolve [4]. Consequently, there is growing interest in identifying alternative programmed cell death modalities that bypass these canonical apoptotic checkpoints [5]. Necroptosis has emerged as a compelling candidate in this regard [6]. Unlike apoptosis, which is immunologically silent, necroptosis is a pro-inflammatory form of regulated necrosis driven by the receptor-interacting protein kinases RIPK1 and RIPK3, along with the pseudokinase MLKL [7, 8]. Activation of this pathway culminates in plasma membrane disruption and the release of damage-associated molecular patterns (DAMPs), potentially promoting anti-tumour immunity [9]. However, in many cancers, the necroptotic machinery is silenced or epigenetically repressed, underscoring the need to understand its upstream regulation.

Traditionally, Beclin 1 has been characterised as a master regulator of autophagy [10]. Beclin 1 exerts its autophagic function through formation of a multiprotein complex with VPS34, a Lipid kinase that generates phosphatidylinositol 3-phosphate (PI3P), and ATG14L, which directs this complex to the endoplasmic reticulum membrane where autophagosome nucleation begins [11]. In this configuration, Beclin 1 serves as a scaffold, VPS34 provides the enzymatic activity required for membrane remodelling, and ATG14L ensures spatial specificity of complex assembly during the early stages of autophagosome biogenesis [10, 11]. Together, this trio orchestrates the initiation of autophagy in response to cellular stress signals [12]. Its tumour suppressor function has been attributed to this role, particularly in maintaining cellular homeostasis and mediating stress responses [13, 14]. However, recent studies Suggest that Beclin 1 May also intersect with necroptotic signalling, beyond its established role in lysosomal degradation. Specifically, Beclin 1 has been implicated in physical and regulatory interactions with RIPK1, suggesting it may exert repressive control over necroptosis [15]. Whether this control is mediated through direct binding, chromatin remodelling, or transcriptional repression remains unresolved [16].

Here, we hypothesize that Beclin 1 not only facilitates autophagy but also suppresses necroptotic cell death at the transcriptional level [17]. To test this, we used small interfering RNA (siRNA) to silence Beclin 1 in HT-29 CRC cells, a model characterised by defective apoptotic signalling [18]. We evaluated changes in mRNA expression of key necroptotic effectors (RIPK1, RIPK3, MLKL) using quantitative reverse transcription Polymerase Chain Reaction (qRT-PCR) and assessed functional activation via western blot analysis of their phosphorylated forms. Our results reveal a novel role for Beclin 1 in transcriptional repression of the necroptotic program, suggesting that its loss may rewire the cell death machinery of CRC cells and sensitise them to immunogenic necrosis. These findings have important implications for therapeutic strategies aimed at reactivating dormant death pathways in apoptosis-resistant tumours.

## Methods

### Cell culture

HT-29 human CRC cells (ATCC^®^ HTB-38™) were cultured in McCoy’s 5 A medium (Gibco, Thermo Fisher Scientific, USA) Supplemented with 10% heat-inactivated fetal bovine serum (FBS; Sigma-Aldrich, USA), 100 units/mL penicillin, and 100 µg/mL streptomycin (Gibco). Cells were Maintained at 37 °C in a humidified atmosphere containing 5% carbon dioxide (CO₂) and Subcultured at 70–80% confluence to ensure optimal growth conditions.

### SiRNA transfection

HT-29 cells were subjected to siRNA targeting the human BECN1 gene. Transfections were performed using Lipofectamine™ RNAiMAX Transfection Reagent (Thermo Fisher Scientific, USA), following the manufacturer’s optimized protocol for high-efficiency delivery in adherent cells. For this, cells were seeded into 6-well tissue culture plates (Corning, USA) at a density of 2 × 10⁵ cells per well in 2 mL of antibiotic-free McCoy’s 5 A medium Supplemented with 10% fetal bovine serum. After 24 h, once cells reached approximately 50–60% confluence, they were transfected with 50 nM of Silencer Select siRNA targeting BECN1 (ID: s16537, Thermo Fisher Scientific, catalog number: 4392420), diluted in Opti-MEM™ Reduced Serum Medium (Thermo Fisher Scientific). Lipofectamine™ RNAiMAX was prepared separately in Opti-MEM and incubated for 5 min at room temperature before combining with the siRNA solution to form siRNA-lipid complexes. The mixture was incubated for 15–20 min at room temperature before being gently introduced to the cells. For negative control experiments, cells were transfected in parallel with Silencer Select Negative Control siRNA #1 (Thermo Fisher Scientific, catalog number: 4390843), a validated scrambled siRNA that does not target any human gene. Cells were incubated under standard culture conditions (37 °C, 5% CO₂) for 48 h post-transfection, after which KD efficiency was assessed by Western blotting and qRT-PCR. All transfections were performed in biological triplicate (*n* = 3) to ensure reproducibility.

### RNA isolation and quantitative reverse transcriptase PCR

Total RNA was isolated from HT-29 cells using the RNeasy Mini Kit (Qiagen, Hilden, Germany) according to the manufacturer’s protocol. Briefly, cells were lysed in RLT buffer containing β-mercaptoethanol, homogenized by pipetting, and passed through a gDNA Eliminator spin column to remove genomic DNA contamination. RNA was eluted in RNase-free water and stored at − 80 °C until use. The concentration and purity of the isolated RNA were assessed using a NanoDrop 2000 spectrophotometer (Thermo Fisher Scientific, USA), ensuring A260/A280 ratios between 1.8 and 2.0. For complementary DNA (cDNA) synthesis, 1 µg of total RNA per sample was reverse-transcribed using the High-Capacity cDNA Reverse Transcription Kit (Applied Biosystems, Foster City, CA, USA) in a 20 µL reaction volume following the manufacturer’s instructions. The thermal cycling conditions included 25 °C for 10 min, 37 °C for 120 min, and 85 °C for 5 min, after which cDNA was stored at − 20 °C. qPCR was conducted using SYBR™ Green PCR Master Mix (Applied Biosystems) on a QuantStudio™ 5 Real-Time PCR System (Thermo Fisher Scientific). Reactions were performed in 10 µL volumes containing 5 µL SYBR Green Master Mix, 0.5 µL of each forward and reverse primer (10 µM), 1 µL cDNA template (diluted 1:5), and 3 µL nuclease-free water. The PCR cycling conditions were; initial denaturation at 95 °C for 10 min, followed by 40 cycles of 95 °C for 15 s and 60 °C for 60 s. Melting curve analysis was included to confirm amplification specificity. The gene-specific primers were used (supplementary data). Each sample was run in technical triplicate, and experiments were repeated in three independent biological replicates (*n* = 3). Gene expression levels were normalized to GAPDH and quantified using the comparative Ct method (2^−ΔΔCt). Data were analysed using QuantStudio™ Design and Analysis Software v2.4.

### Protein extraction and Western blotting

Cells were lysed in RIPA buffer (Thermo Fisher Scientific, catalog number: 89900) with protease and phosphatase inhibitors (Roche, 04906845001). Protein concentration was determined using the Pierce™ BCA Protein Assay Kit. Equal amounts of protein (30 µg) were resolved on 10% SDS-PAGE and transferred to PVDF membranes (Millipore). Membranes were blocked with 5% non-fat dry milk in TBST and probed overnight at 4 °C with the following primary antibodies: anti-Beclin 1 antibody (ab210498, EPR20473), anti-RIP antibody (ab300617, EPR2488385), anti-RIP3 (phospho s227, ab209384, EPR9627), anti-MLKL (phospho S345, ab196436, EPR9515 (2)), anti-beta actin (ab8226, Abcam). Blots were incubated with HRP-conjugated secondary antibodies (CST), visualized using ECL Western Blotting Substrate (Thermo Fisher), and imaged with a ChemiDoc MP system (Bio-Rad). Densitometry was performed using ImageJ.

### Statistical analysis

All experiments were independently repeated at least three times, with results showing the mean and error bars highlighting the standard error of the mean (SEM), meaning the standard deviation (SD) divided by the square root of the number of repeats, which shows how the mean of the population can be estimated from the individual sample displayed. Standard deviations are also available in Online Resource 1. Statistical significance was assessed using unpaired one-tailed Student’s t-tests assuming unequal variances. P-values < 0.05 were considered statistically significant (Online resource 1).

## Results

This study reveals a previously underappreciated role of Beclin 1 in maintaining necroptotic silencing within CRC cells [19]. Through siRNA-mediated silencing of *BECN1*, we demonstrate that Beclin 1 constrains the expression of core necroptosis mediators, *RIPK1*, *RIPK3*, and *MLKL*, at the transcriptional level [20], while functionally limiting downstream phosphorylation events that would otherwise trigger regulated necrosis [21]. Its loss creates a death-permissive molecular landscape that leaves CRC cells transcriptionally primed for necroptosis, even in the absence of extrinsic stimuli [22]. Importantly, this transcriptional reprogramming occurs in HT-29 cells, a model of apoptosis resistance [23], highlighting Beclin 1 suppression as a potential strategy to sensitise tumours that are otherwise refractory to caspase-dependent death [24]. These findings position Beclin 1 not only as an autophagy modulator but as a dual-role Suppressor of immunogenic necroptosis, implicating it as a central node in death pathway coordination. Targeting Beclin 1 in combination with necroptosis inducers, death ligands, or immunotherapy agents could provide a novel therapeutic axis to exploit in CRC and other apoptosis-resistant malignancies [25]. Future studies investigating the transcriptional Machinery downstream of Beclin 1 loss will be critical to further define this regulatory circuit and translate these findings into therapeutic benefit.

### Efficient Beclin 1 silencing in HT-29 cells enables investigation of cell death reprogramming

**Fig. 1:**
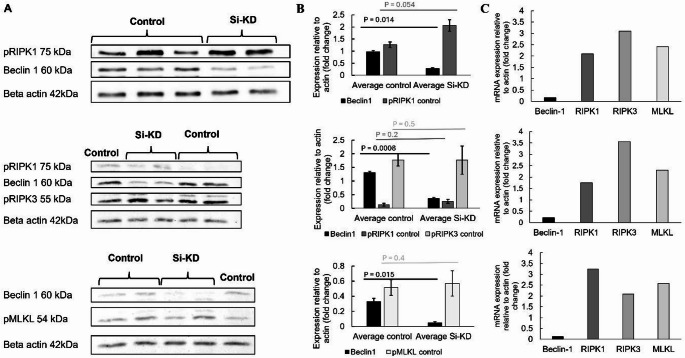
Beclin 1 knockdown upregulates necroptotic mediators in HT-29 cells. (**A**) Western blot images showing expression of Beclin 1, phosphorylated (‘p’) pRIPK1, pRIPK3 and pMLKL in HT-29 cells transfected with either control siRNA or BECN1 siRNA Beclin1 knockdown (Si-KD) for 48 h. β-actin served as a loading control. (**B**) Quantitative densitometry analysis of Beclin 1, pRIPK1, and pMLKL protein levels normalized to β-actin. Data are expressed as fold change relative to scrambled control (mean ± SEM, *n* = 3). *p* < 0.05, *p* < 0.01 (unpaired t-test). (**C**) qRT-PCR analysis showing increased mRNA expression of RIPK1, RIPK3, and MLKL upon BECN1 KD. Values were normalized to GAPDH and are presented as fold change relative to control. Each bar represents the mean ± SEM (*n* = 3). Statistical significance: *p* < 0.05, *p < 0.01

To investigate the role of Beclin 1 in modulating non-apoptotic cell death pathways, HT-29 CRC cells were transfected with siRNA targeting *BECN1* [19, 21]. Immunoblotting confirmed efficient KD of Beclin 1, with levels reduced by approximately 50–85% across three independent biological replicates (Fig. 1A, b; *P* < 0.02, unpaired *t*-test). β-actin was used as a loading control. No evidence of cytotoxicity or morphological disruption was observed within 48 h post-transfection, validating the system for downstream interrogation of necroptotic signalling components.

### Beclin 1 silencing induces transcriptional upregulation of Necroptosis-related genes

qRT-PCR revealed that KD of Beclin 1 led to marked increases in the mRNA levels of key necroptotic mediators (Fig. 1C). Specifically, *RIPK1*, *RIPK3*, and *MLKL* expression levels were elevated by 2.36-fold (*p* < 0.01), 2.92-fold (*p* < 0.005), and 2.42-fold (*p* < 0.01), respectively, compared to scrambled siRNA controls. The consistency across all three genes Suggests a coordinated transcriptional de-repression event. Notably, this transcriptional induction occurred in the absence of external death Ligands or chemical stressors, implicating Beclin 1 in a previously unappreciated suppressive role on the basal transcriptional activity of the necroptotic axis.

### Functional activation of necroptosis pathway is initiated but incomplete

To assess whether the observed transcriptional changes translated into functional priming of necroptosis, we next analysed protein phosphorylation status. Western blotting demonstrated a modest yet consistent increase in pRIPK1 (Ser166) by ~ 80% following Beclin 1 KD (Fig. 1A, B). Similarly, pRIPK2 increased 12%, and pMLKL (Ser358) was elevated by ~ 5%. In contrast, pRIPK3 was not detected to increase. Although phosphorylation levels are not indicative of full necroptotic execution, these changes suggest a primed state, wherein the core necrosome components are both transcriptionally abundant and poised for activation. Of note, pRIPK3 levels were not increased, consistent with a model in which Beclin 1 silencing alone establishes a permissive, but not fully activated, necroptotic landscape. This aligns with previous observations that full pathway execution often requires additional extrinsic stimuli such as TNF-α, caspase inhibition, or oxidative stress.

## Discussion

Tumour cell resistance to apoptosis remains a major obstacle to effective cancer therapy [26]. Several papers have shown that Beclin 1’s coordination of other proteins like USP19, LC3, and Rictor can increase hikelihood of metastasis and drug resistance [27, 28]. Previous studies have also established the canonical role of Beclin 1 in the initiation of autophagy [12], where it coordinates phosphatidylinositol 3-kinase complex formation and autophagosome biogenesis [29]. Through this role, therapeutics have been suggested [30]. For example, carnosine has been proposed to suppress human CRC cell proliferation by inducing necroptosis and autophagy and reducing angiogenesis. Beclin-1 and its contol of autophagy were highlighted as a key factor in carnosine’s Success as Beclin 1 mRNA was significantly increased by 137–141% in HCT-116 cells treated with 5, 10 or 15 mM carnosine [31]. This reveals the contradictory troles of Beclin 1 in CRC, whereby autophagy is naturally used by normal cells to protect from malignancy by removing aggregated proteins, while reducing reactive oxygen species and DNA damage [32]. However, Beclin 1 medated autophagy also supports metastatic transformation by inhibiting cell death, while increasing nutrient availability, metabolism, cell growth and drug resistance [32]. Therefore Beclin 1 and its canonical role in autophagy organisation is already understood as a central and critical player in CRC.

However, emerging evidence has implicated Beclin 1 in additional non-canonical roles, including its interactions with death domain-containing proteins and chromatin modifiers [33]. Our data extend these findings by Suggesting that Beclin 1 actively maintains necroptotic quiescence as a critical molecular barrier to necroptotic reprogramming in CRC HT-29 cells by indirectly constraining the transcriptional availability of necroptotic effectors including RIPK1, RIPK3, and MLKL. The upregulation of transcripts following Beclin 1 depletion suggests de-repression of gene programs that are otherwise held in check under normal conditions and suggests another way Beclin1 shields CRC cells from immunogenic cell death.

Interestingly, despite strong transcriptional induction, phosphorylated RIPK1 and MLKL proteins increased only modestly in the absence of extrinsic necroptotic triggers. This Suggests that Beclin 1 silencing creates a primed, pre-activated state, where the machinery of necroptosis is present but not fully engaged. Such a poised configuration may serve as a latent vulnerability that could be exploited using death-inducing ligands (e.g., TNF-α) [34], caspase inhibitors (e.g., z-VAD-fmk) [35], or necroptosis activators (e.g., Smac mimetics) [36]. This is particularly significant given the immunogenic nature of necroptosis; triggering it in situ within apoptosis-resistant tumours may enhance antigen release and dendritic cell recruitment, effectively converting “cold” tumours into immunologically “hot” microenvironments [37].

Our data Suggests that Beclin 1 presence within cells may suppress transcription of RIPK1, RIPK3, and MLKL as upon Beclin 1 deletion transcription of these necroptotic mRNAs increases. However, the exact mechanism remains to be elucidated. It is possible but unlikely that Beclin 1 itself is a transcription factor, which directly inhibits necroptotic factor transcription. Beclin 1 contains a leucine-rich nuclear export signal motif which when mutated prevents Beclin 1 from fulfilling its role in the cells nutrient deprivation-induced autophagy and tumor supressive functions in human breast carcinoma (MCF7) cells. Therefore, most Beclin 1 is exported from the nucleus and loss of its activity in the cytoplasm May contribute to tumorigenesis. While there is some evidence for nuclear localisation of Beclin 1, where it may associate with and regulate DNA double-strand break repair proteins, causing increased DNA repair [38]. Overall, it appears that Beclin 1 is designed for intricate and dynamic protein-protein interactions, but not protein-DNA interactions. As a result, it is most Likely Beclin 1’s effect on RIPK1 and MLKL transcription is mediated indirectly. Beclin 1 is Such a central part of autophagy regulation, its absence has a Major impact on many signalling pathways Like autophagy initiation, receptor trafficking and stress response pathways which affect the transcription of Many genes. One plausible hypothesis is that Beclin 1 regulates transcriptional repressors or chromatin remodelling factors, potentially via its known interaction with histone deacetylases or methylation-associated proteins [39]. Beclin 1 is known to have key relationships with transcription factors, For example Sex-determining region Y-box2 (SOX2). Both form part of the previously explored carcinogenic SOX2-β-catenin/Beclin1-ABCC2 axis whereby SOX2 transcriptionally activates Beclin 1 inducing autophagy and a malignant phenotype [40]. Alternatively, Beclin 1 May act through miRNA-mediated control of necroptotic mRNAs, as Beclin 1 has been previously linked to post-transcriptional regulation in cancer cells [41]. Regardless of the exact mechanism, our findings Suggest that loss of Beclin 1 is not a neutral autophagic deficit but a profound reconfiguration of cell death potential, redirecting CRC cells from silent, caspase-dependent apoptosis toward inflammatory, caspase-independent necrosis.

Our study is not without Limitations. While we establish a Link between Beclin 1 KD and necroptotic priming in HT-29 cells, further validation across additional CRC models with distinct mutational backgrounds is warranted. To expand on this study in the future, another identical MSS/KRAS-mutant CRC cell line like the widely used DLD-1 could be tested as a positive control, assuming the de-repressive effect on necroptotic factors would remain the same [42]. Meanwhile, other CRC lines with different aspects of wnt signalling activation could be explored. For example, RKO cells, which are more p53-competent, but have a different Wnt context and MSS/KRAS-WT cells which would provide a counterpoint to the MSS/KRAS-mutant line used in this study [43]. Finaly, a wnt-high secretory model like LS174T could be explored to test the effects in the wnt active model. Application of our study in a panel of these other CRC cell lines would pave the way for investigation within in-vivo models before potential therapeutic exploration. Moreover, the modest levels of pRIPK1 and pMLKL observed suggest that full necroptotic execution likely requires co-stimulatory input. Functional assays, such as propidium iodide uptake, Annexin V/PI flow cytometry [41], or MLKL translocation imaging [44], would provide definitive evidence of necroptotic death execution. Future studies could also integrate chromatin immunoprecipitation (ChIP-seq) and transcriptomic profiling to elucidate the direct transcriptional targets of Beclin 1 and identify cofactors involved in necroptosis repression [26].

## Conclusions

In Summary, our data Suggests a dual role for Beclin 1 in CRC: as an autophagic initiator and as a suppressor of necroptotic transcription. Its depletion reprograms CRC cells into a state of heightened susceptibility to necroptosis, offering a potential therapeutic avenue for overcoming apoptosis resistance. These findings open new directions in the design of combination therapies that exploit necroptotic sensitisation, for example, pairing autophagy inhibitors with necroptosis inducers or immune checkpoint blockade to synergistically dismantle tumour survival mechanisms. As immunotherapies evolve, manipulating the balance between survival and death pathways such as autophagy and necroptosis may offer the key to overcoming therapeutic resistance in colorectal and other solid tumours.

## Supplementary Information

Below is the link to the electronic supplementary material.

Supplementary Material 1

Supplementary Material 2

Supplementary Material 3

## Data Availability

No datasets were generated or analysed during the current study.
