# Supplementary material for: Loss of Beclin 1 primes colorectal cancer cells for Immunogenic necroptosis via transcriptional de-repression of RIPK1/RIPK3/MLKL axis
Source: Mol Biol Rep. 2025 Sep 19;52(1):925. doi: 10.1007/s11033-025-11037-6 (PMC12449333; doi:10.1007/s11033-025-11037-6)
Supplement: Supplementary file 1 — Supplementary Material 1 [file 11033_2025_11037_MOESM1_ESM.pptx]

## Slide 1
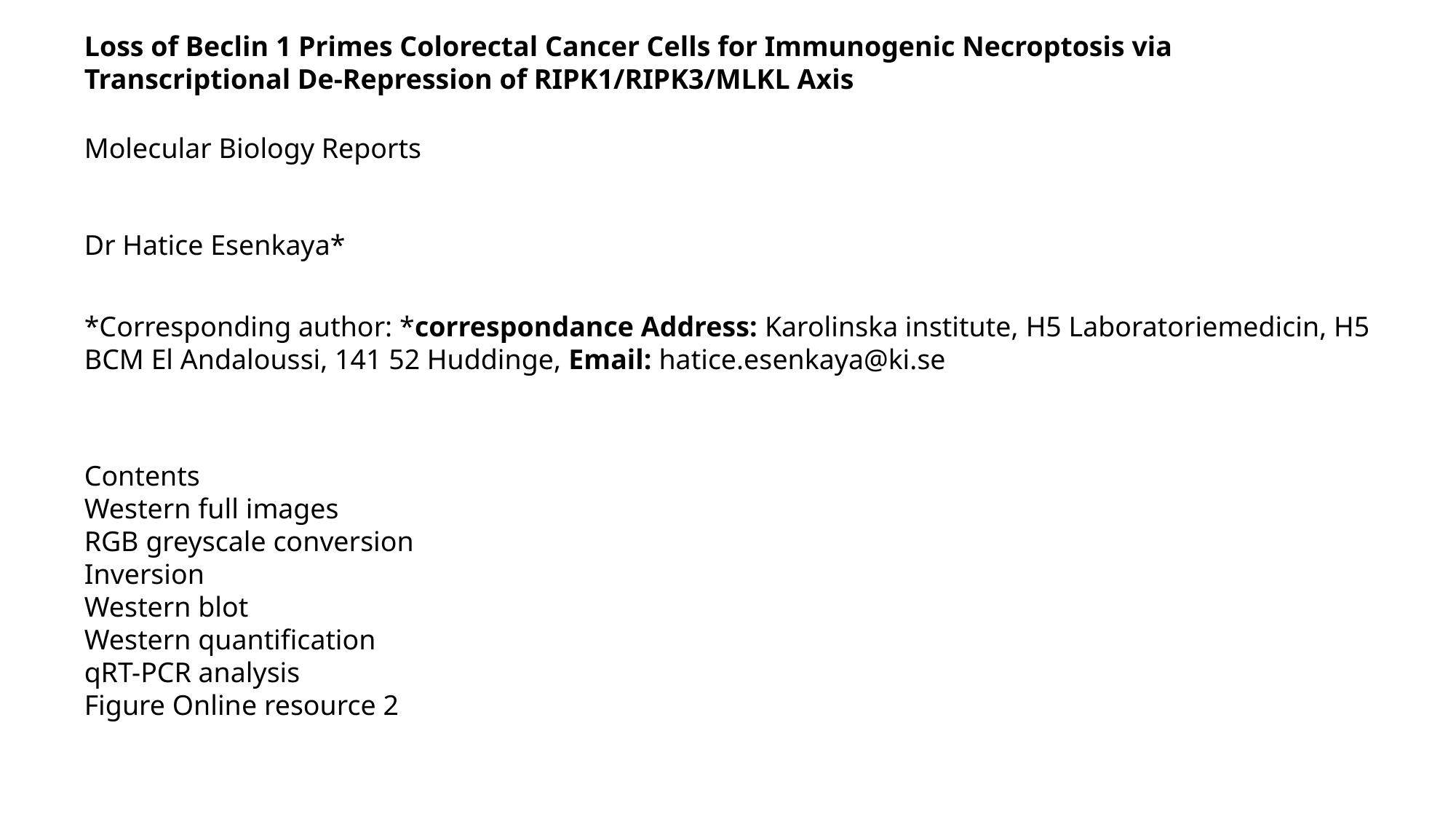

Loss of Beclin 1 Primes Colorectal Cancer Cells for Immunogenic Necroptosis via Transcriptional De-Repression of RIPK1/RIPK3/MLKL Axis
Molecular Biology Reports
Dr Hatice Esenkaya*
*Corresponding author: *correspondance Address: Karolinska institute, H5 Laboratoriemedicin, H5 BCM El Andaloussi, 141 52 Huddinge, Email: hatice.esenkaya@ki.se
Contents
Western full images
RGB greyscale conversion
Inversion
Western blot
Western quantification
qRT-PCR analysis
Figure Online resource 2

## Slide 2
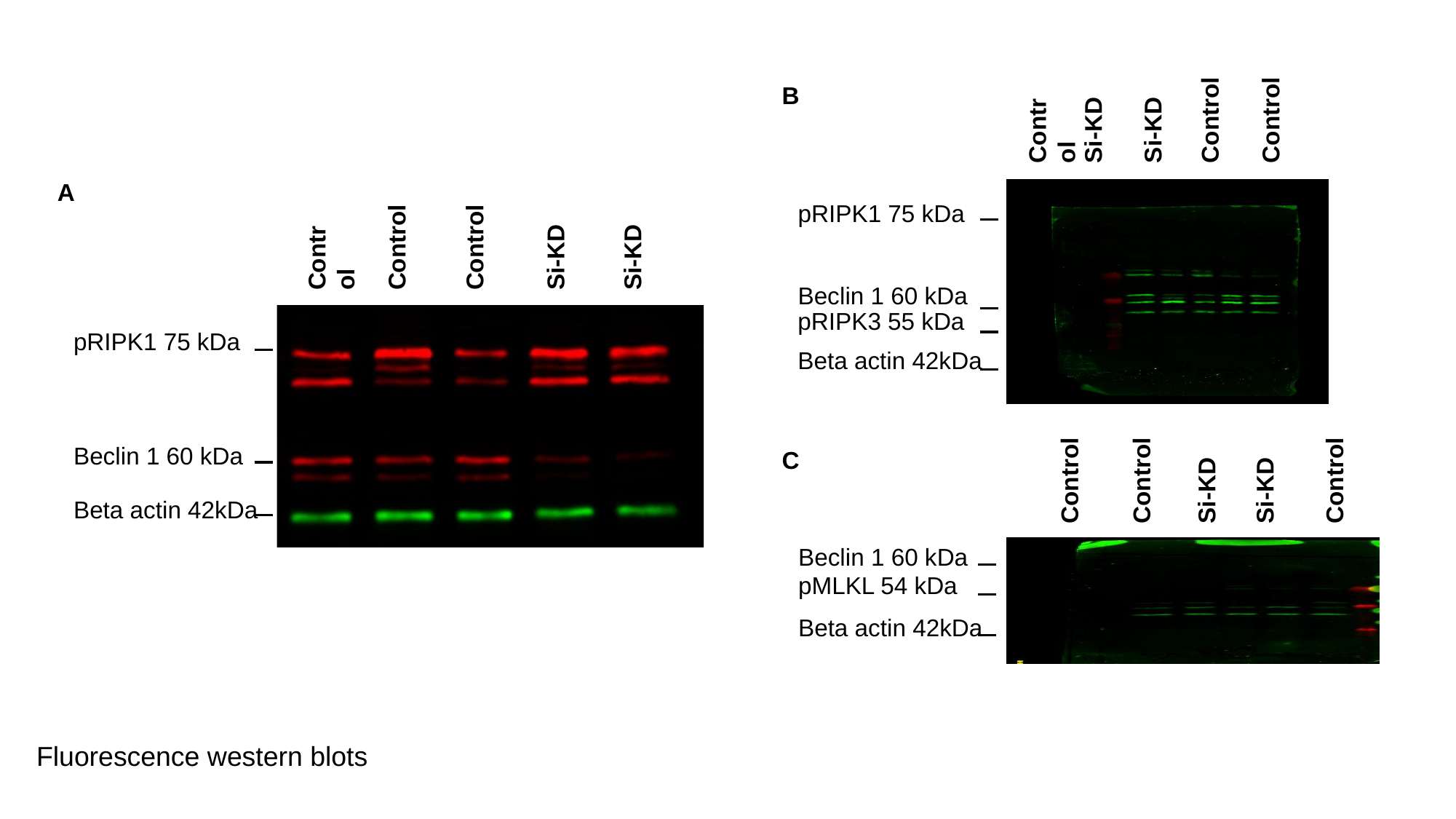

Si-KD
Si-KD
Control
Control
B
Control
pRIPK1 75 kDa
A
Beclin 1 60 kDa
Control
Control
Control
Si-KD
Si-KD
pRIPK3 55 kDa
pRIPK1 75 kDa
Beta actin 42kDa
Beclin 1 60 kDa
Si-KD
Si-KD
Control
Control
Beta actin 42kDa
Control
C
Beclin 1 60 kDa
pMLKL 54 kDa
Beta actin 42kDa
Fluorescence western blots

## Slide 3
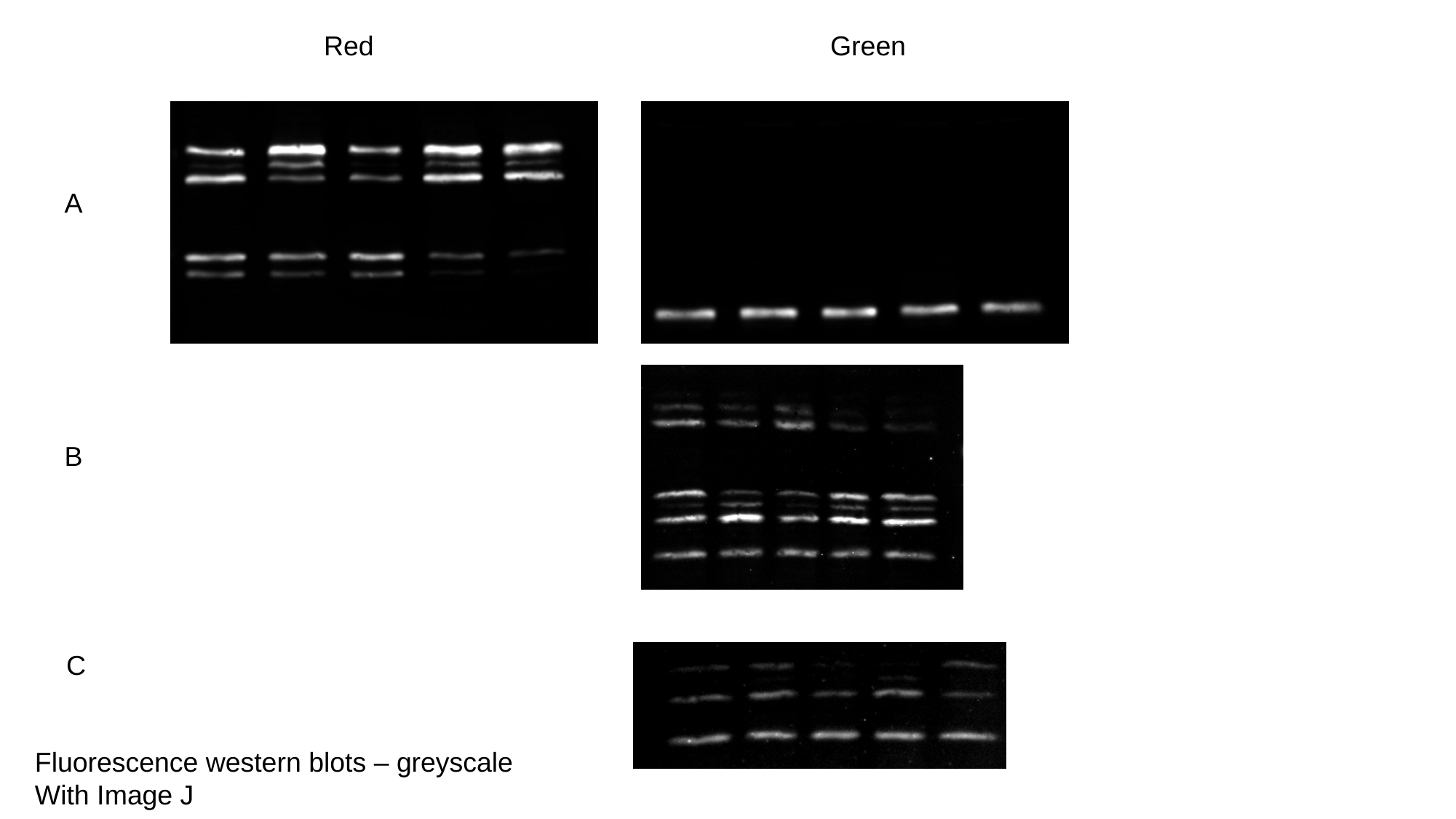

Red
Green
A
B
C
Fluorescence western blots – greyscale
With Image J

## Slide 4
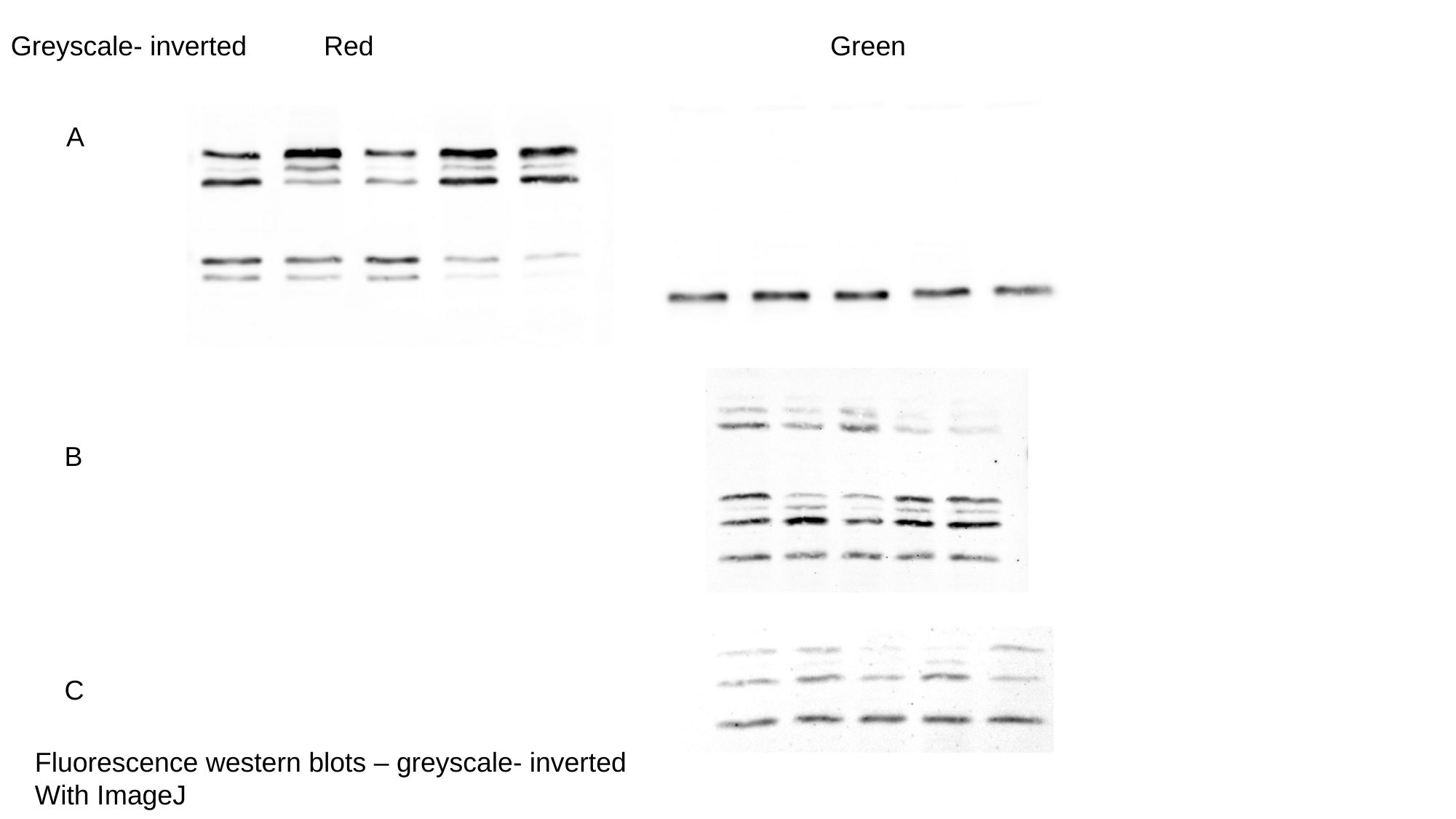

Greyscale- inverted
Red
Green
A
B
C
Fluorescence western blots – greyscale- inverted
With ImageJ

## Slide 5
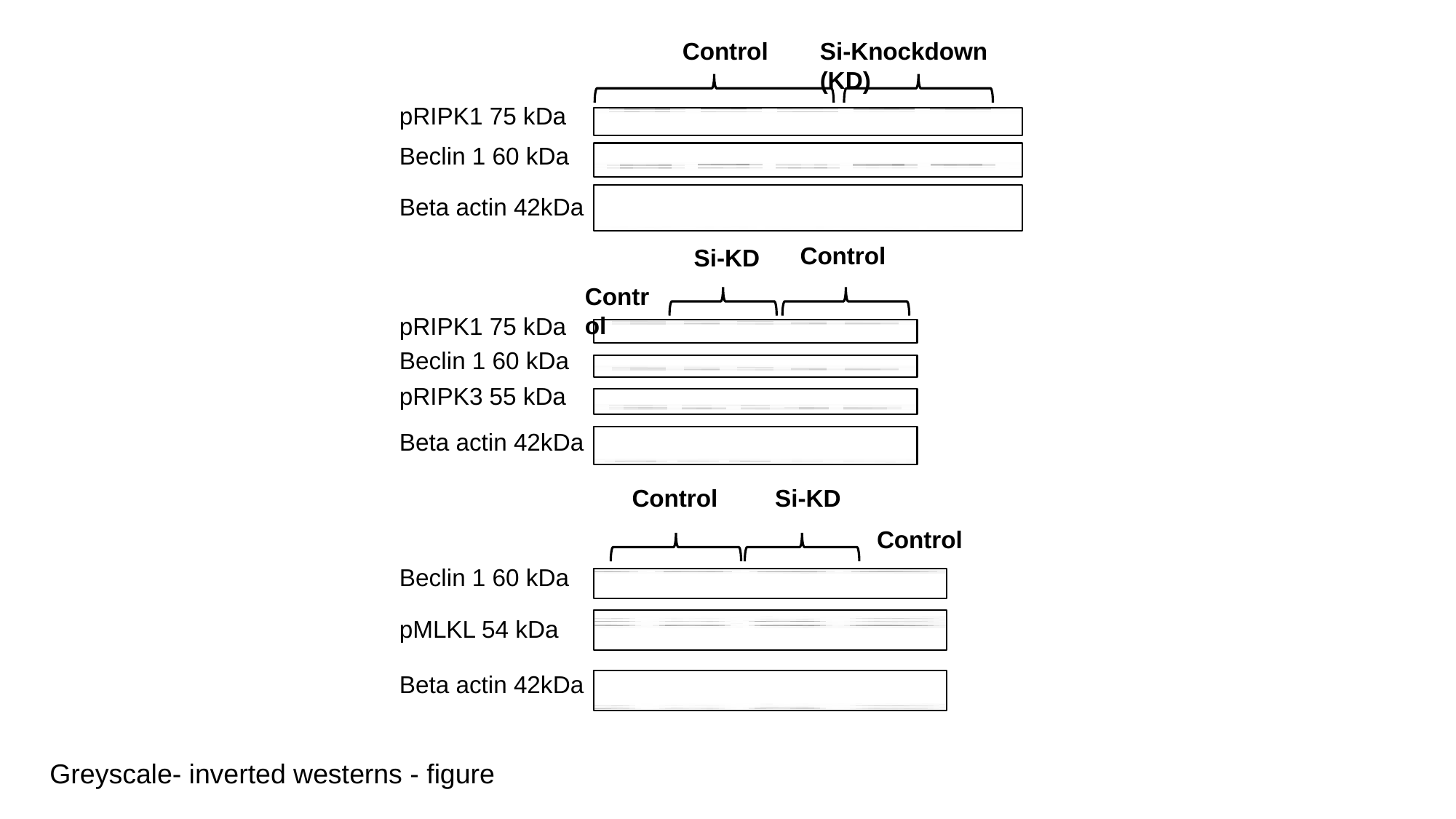

Si-Knockdown (KD)
Control
pRIPK1 75 kDa
Beclin 1 60 kDa
Beta actin 42kDa
Control
Si-KD
pRIPK1 75 kDa
Control
Beclin 1 60 kDa
pRIPK3 55 kDa
Beta actin 42kDa
Control
Si-KD
Beclin 1 60 kDa
Control
pMLKL 54 kDa
Beta actin 42kDa
Greyscale- inverted westerns - figure

## Slide 6
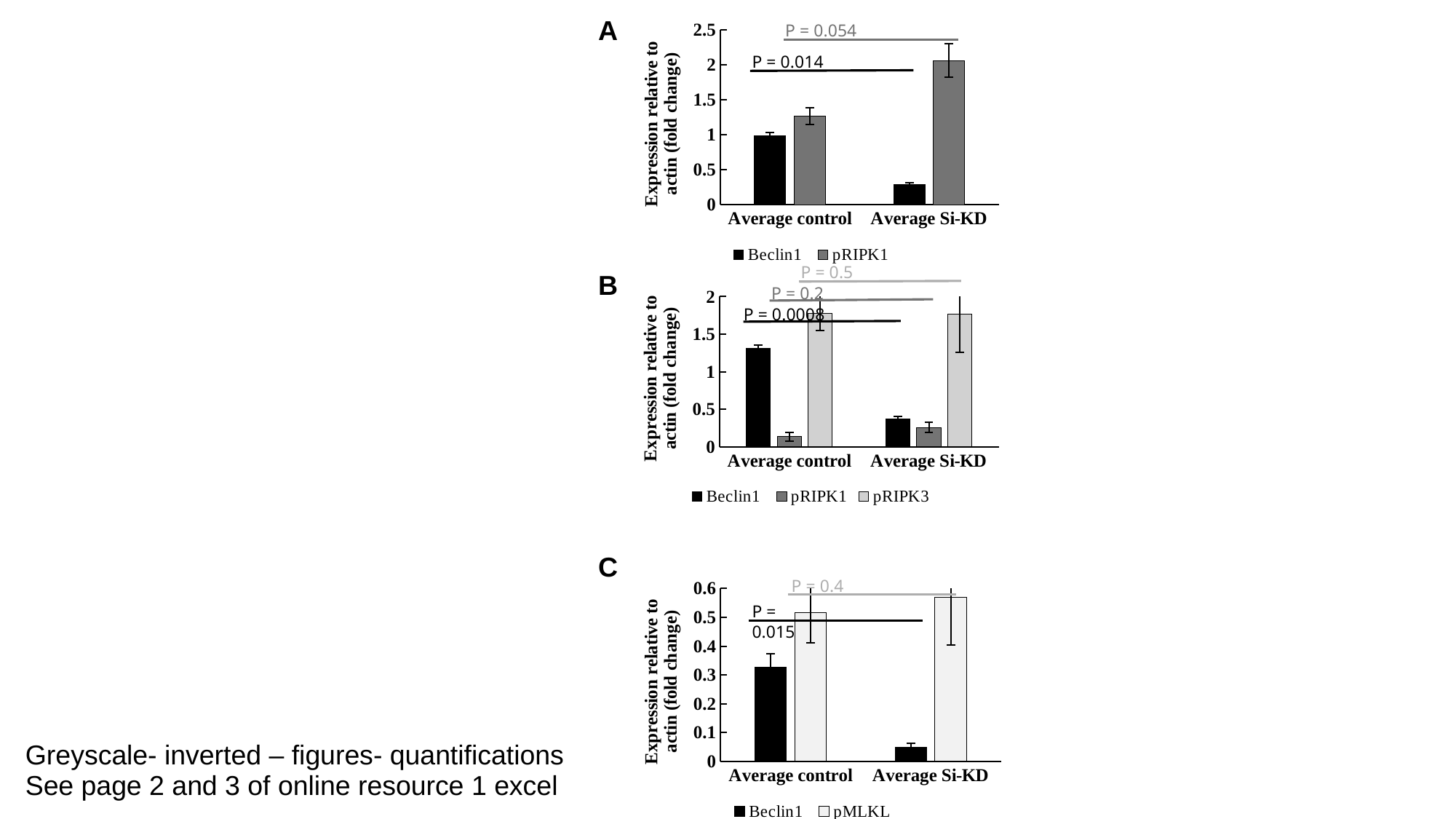

A
### Chart
| Category | Beclin1 | pRIPK1 |
|---|---|---|
| Average control | 0.9806391364554737 | 1.2631739218003255 |
| Average Si-KD | 0.28338593043103416 | 2.0609102549084426 |P = 0.054
P = 0.014
P = 0.5
P = 0.2
### Chart
| Category | Beclin1 | pRIPK1 | pRIPK3 |
|---|---|---|---|
| Average control | 1.3136134868822378 | 0.1339972656865274 | 1.779096933454407 |
| Average Si-KD | 0.36784064889398216 | 0.2576067593324884 | 1.767966790320732 |P = 0.0008
B
C
P = 0.4
### Chart
| Category | Beclin1 | pMLKL |
|---|---|---|
| Average control | 0.326583726816662 | 0.515300092381071 |
| Average Si-KD | 0.04971976109504216 | 0.5696808277788015 |P = 0.015
Greyscale- inverted – figures- quantifications
See page 2 and 3 of online resource 1 excel

## Slide 7
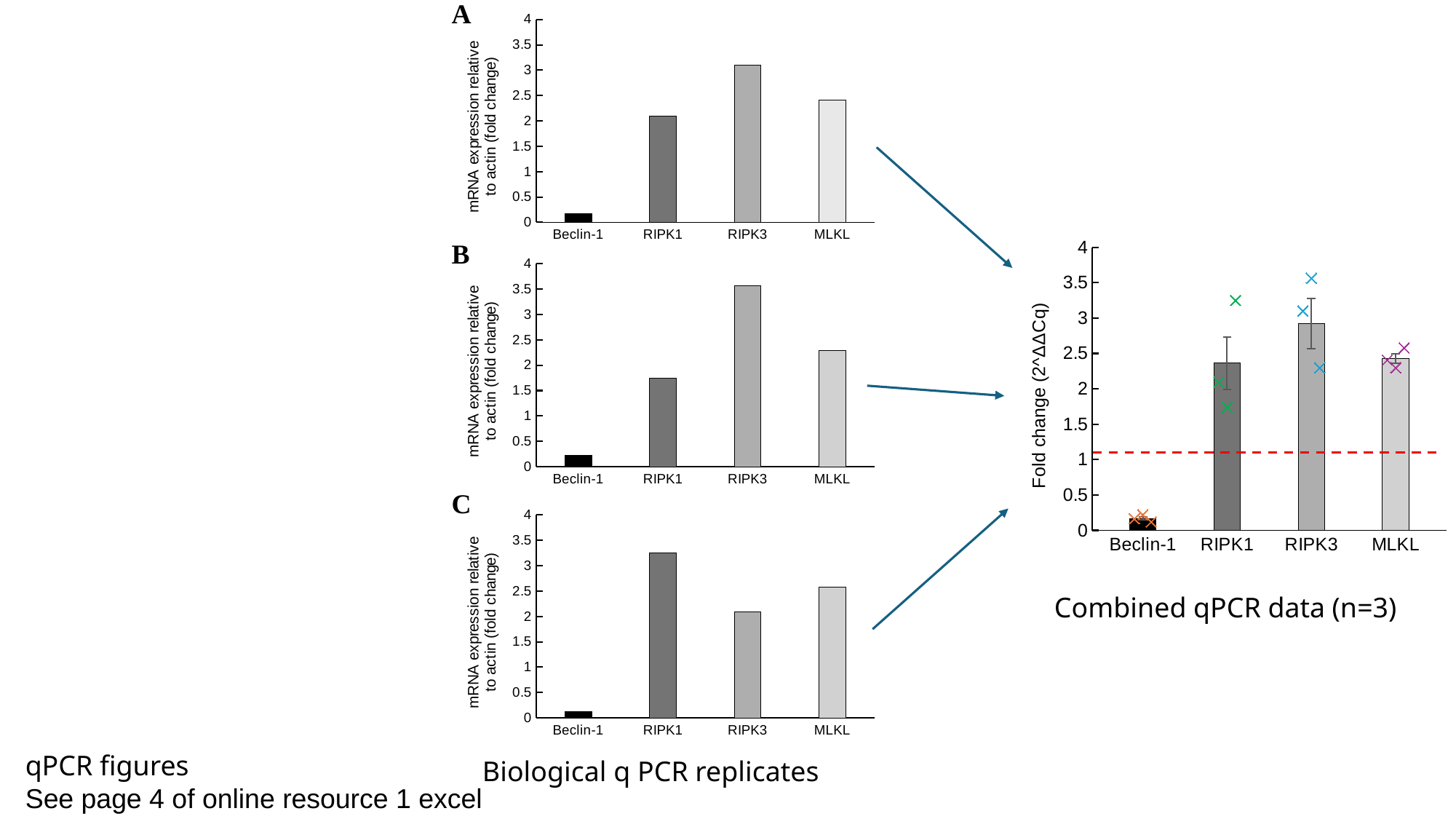

A
### Chart
| Category | Average fold-change |
|---|---|
| Beclin-1 | 0.16493848884661244 |
| RIPK1 | 2.0945882456412632 |
| RIPK3 | 3.1022895236674755 |
| MLKL | 2.4060500721642373 |B
[unsupported chart]
### Chart
| Category | Average fold-change |
|---|---|
| Beclin-1 | 0.2227246795350852 |
| RIPK1 | 1.7411011265922491 |
| RIPK3 | 3.5635948725613456 |
| MLKL | 2.297396709994063 |C
### Chart
| Category | Average fold-change |
|---|---|
| Beclin-1 | 0.11935520048880237 |
| RIPK1 | 3.249009585424956 |
| RIPK3 | 2.0945882456412583 |
| MLKL | 2.5787406168791653 |Combined qPCR data (n=3)
qPCR figures
Biological q PCR replicates
See page 4 of online resource 1 excel

## Slide 8
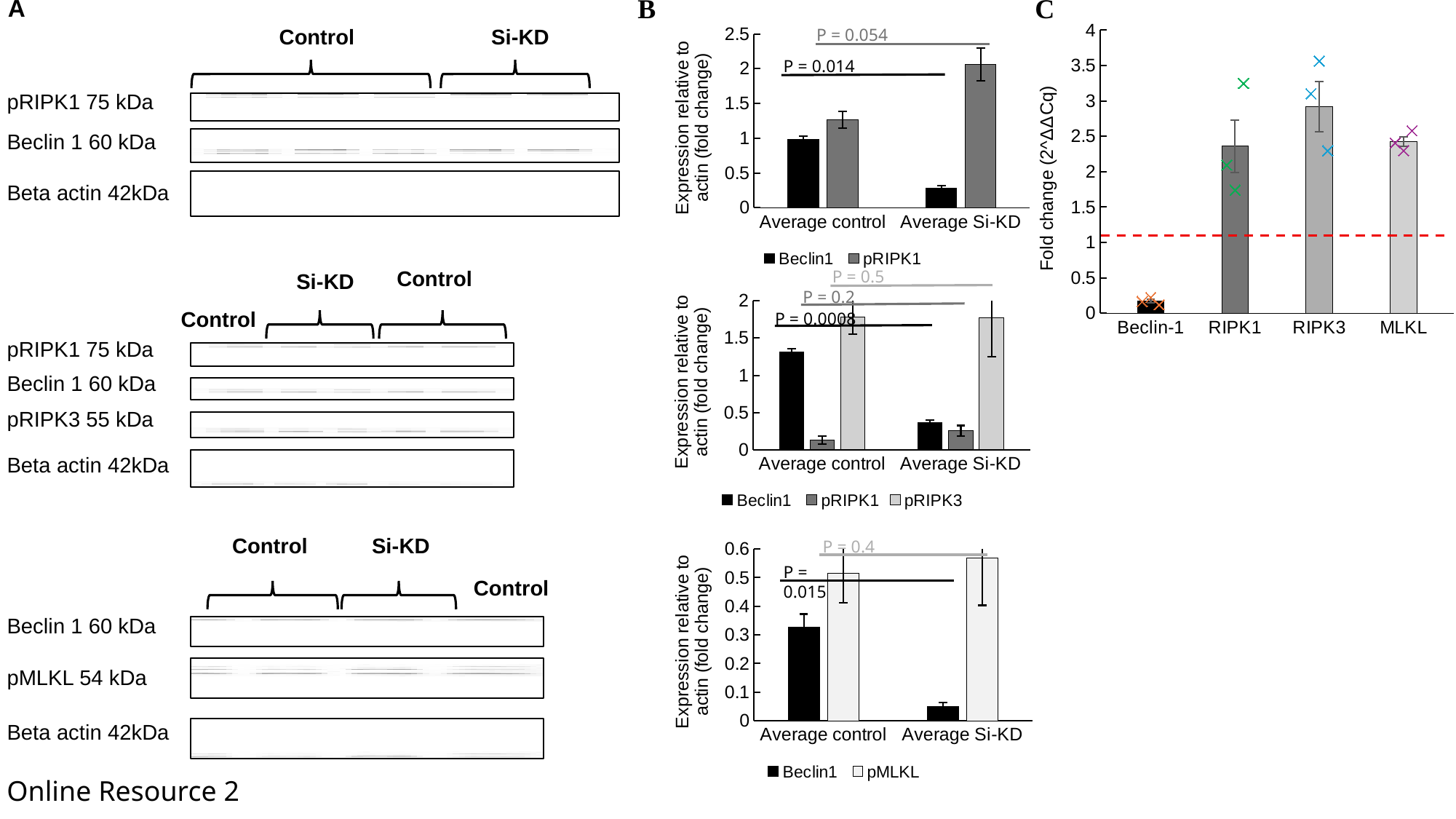

A
B
C
[unsupported chart]
Si-KD
Control
pRIPK1 75 kDa
Beclin 1 60 kDa
Beta actin 42kDa
P = 0.054
### Chart
| Category | Beclin1 | pRIPK1 |
|---|---|---|
| Average control | 0.9806391364554737 | 1.2631739218003255 |
| Average Si-KD | 0.28338593043103416 | 2.0609102549084426 |P = 0.014
Control
Si-KD
pRIPK1 75 kDa
Control
Beclin 1 60 kDa
pRIPK3 55 kDa
Beta actin 42kDa
P = 0.5
P = 0.2
### Chart
| Category | Beclin1 | pRIPK1 | pRIPK3 |
|---|---|---|---|
| Average control | 1.3136134868822378 | 0.1339972656865274 | 1.779096933454407 |
| Average Si-KD | 0.36784064889398216 | 0.2576067593324884 | 1.767966790320732 |P = 0.0008
Control
Si-KD
Beclin 1 60 kDa
Control
pMLKL 54 kDa
Beta actin 42kDa
P = 0.4
### Chart
| Category | Beclin1 | pMLKL |
|---|---|---|
| Average control | 0.326583726816662 | 0.515300092381071 |
| Average Si-KD | 0.04971976109504216 | 0.5696808277788015 |P = 0.015
Online Resource 2

## Slide 9
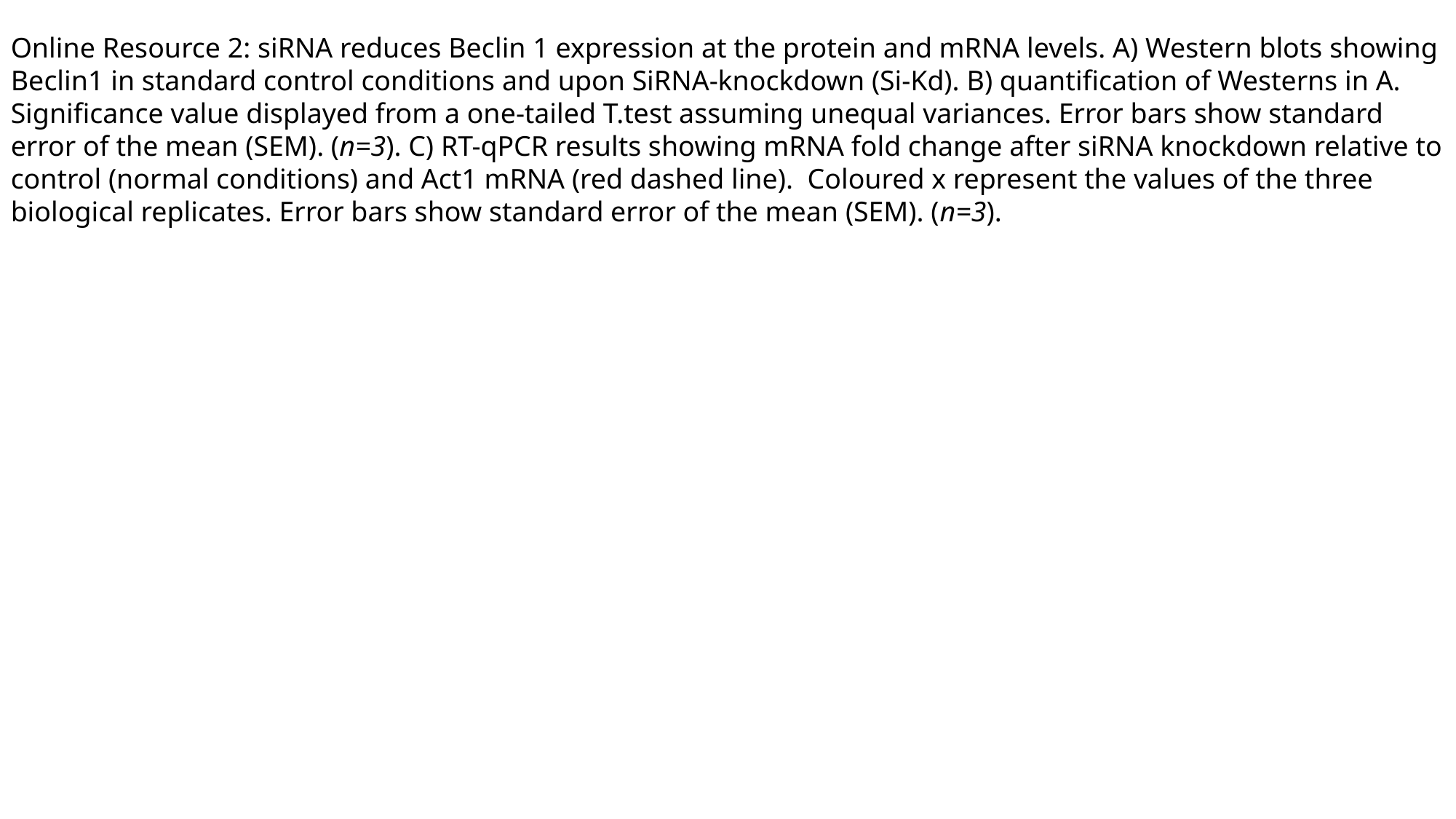

Online Resource 2: siRNA reduces Beclin 1 expression at the protein and mRNA levels. A) Western blots showing Beclin1 in standard control conditions and upon SiRNA-knockdown (Si-Kd). B) quantification of Westerns in A. Significance value displayed from a one-tailed T.test assuming unequal variances. Error bars show standard error of the mean (SEM). (n=3). C) RT-qPCR results showing mRNA fold change after siRNA knockdown relative to control (normal conditions) and Act1 mRNA (red dashed line). Coloured x represent the values of the three biological replicates. Error bars show standard error of the mean (SEM). (n=3).
