# Supplementary material for: Loss of Beclin 1 primes colorectal cancer cells for Immunogenic necroptosis via transcriptional de-repression of RIPK1/RIPK3/MLKL axis
Source: Mol Biol Rep. 2025 Sep 19;52(1):925. doi: 10.1007/s11033-025-11037-6 (PMC12449333; doi:10.1007/s11033-025-11037-6)
Supplement: Supplementary file 3 — Supplementary Material 3 [file 11033_2025_11037_MOESM3_ESM.pptx]

## Slide 1
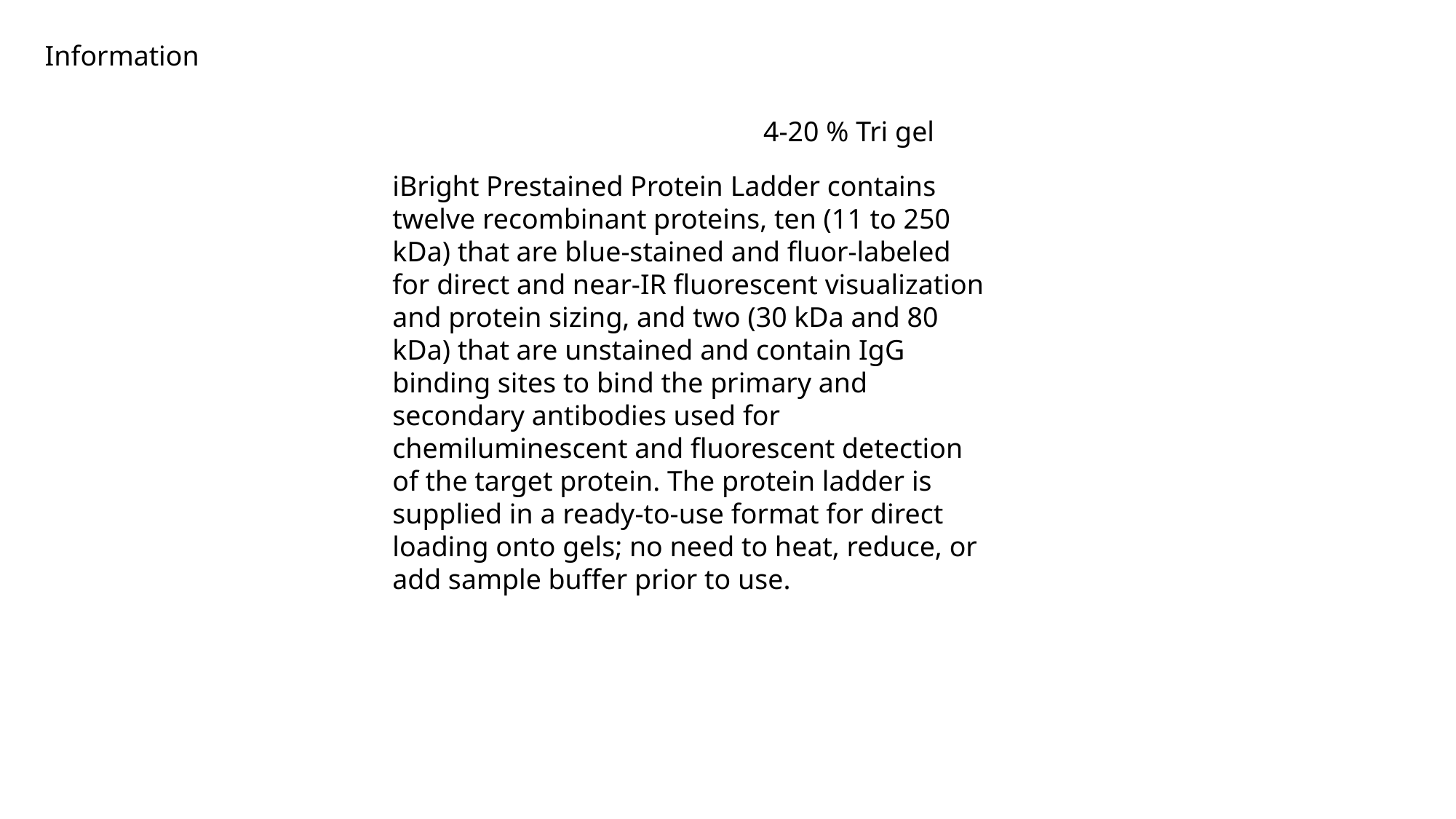

Information
4-20 % Tri gel
iBright Prestained Protein Ladder contains twelve recombinant proteins, ten (11 to 250 kDa) that are blue-stained and fluor-labeled for direct and near-IR fluorescent visualization and protein sizing, and two (30 kDa and 80 kDa) that are unstained and contain IgG binding sites to bind the primary and secondary antibodies used for chemiluminescent and fluorescent detection of the target protein. The protein ladder is supplied in a ready-to-use format for direct loading onto gels; no need to heat, reduce, or add sample buffer prior to use.

## Slide 2
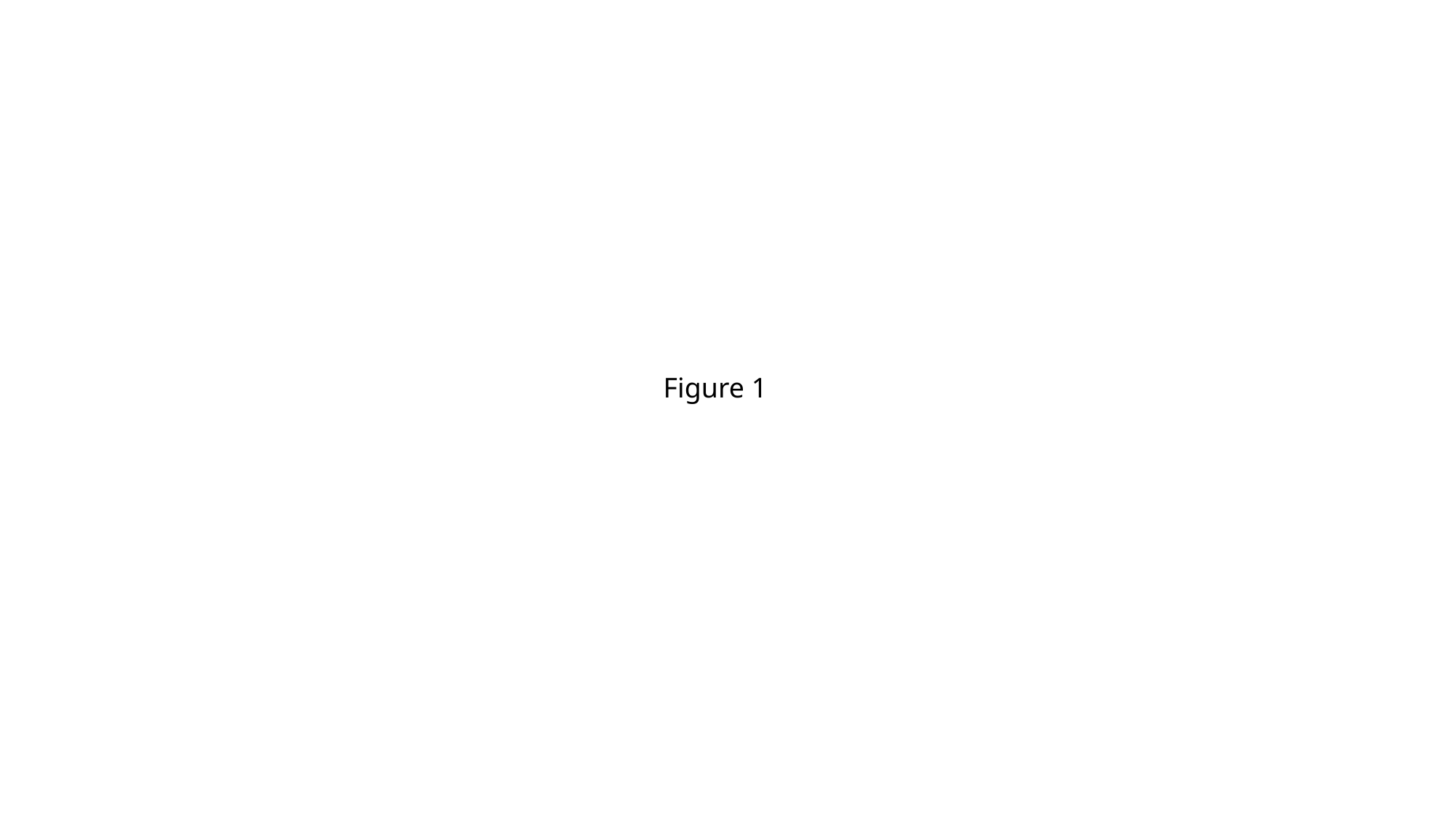

Figure 1

## Slide 3
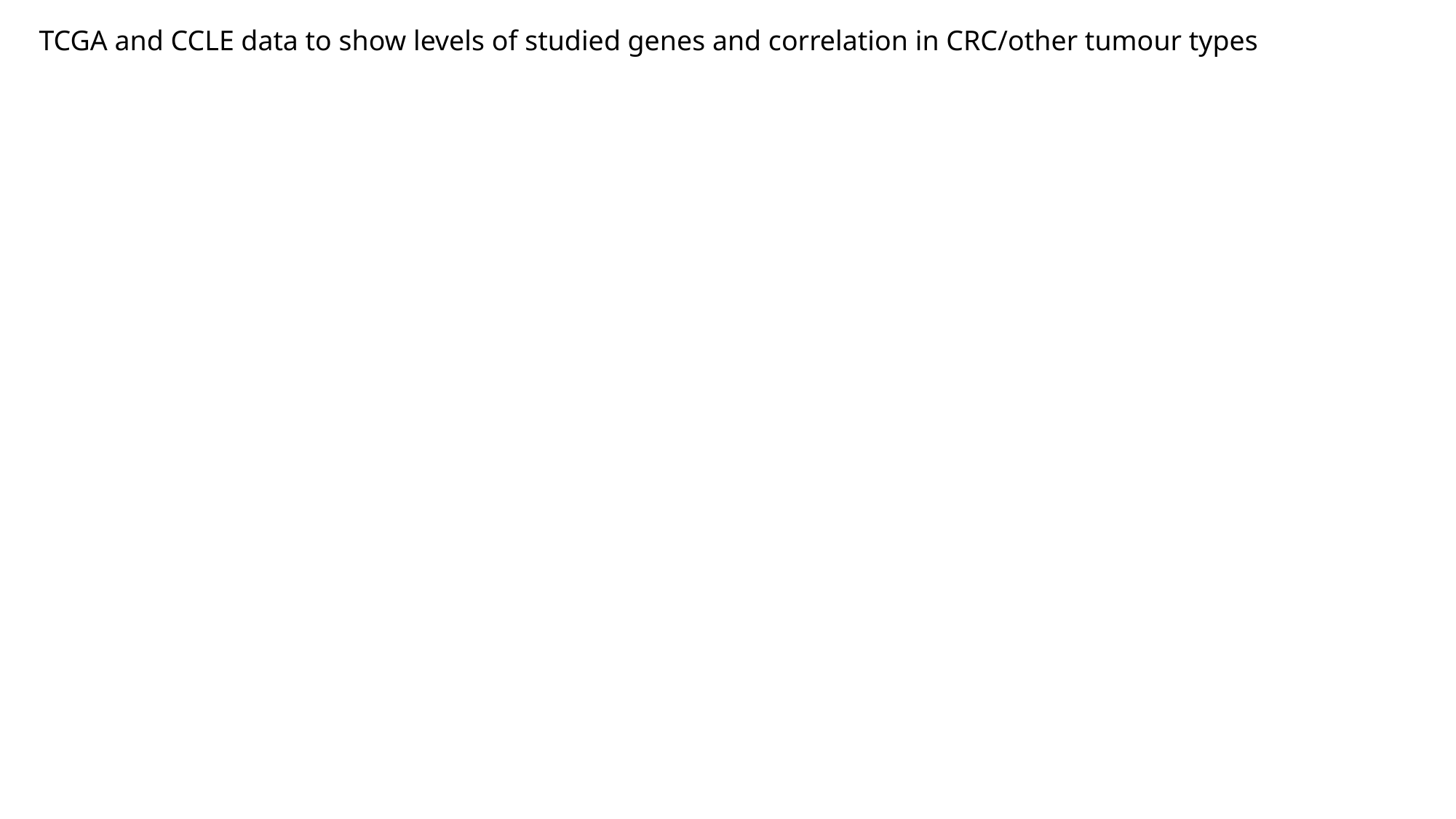

TCGA and CCLE data to show levels of studied genes and correlation in CRC/other tumour types

## Slide 4
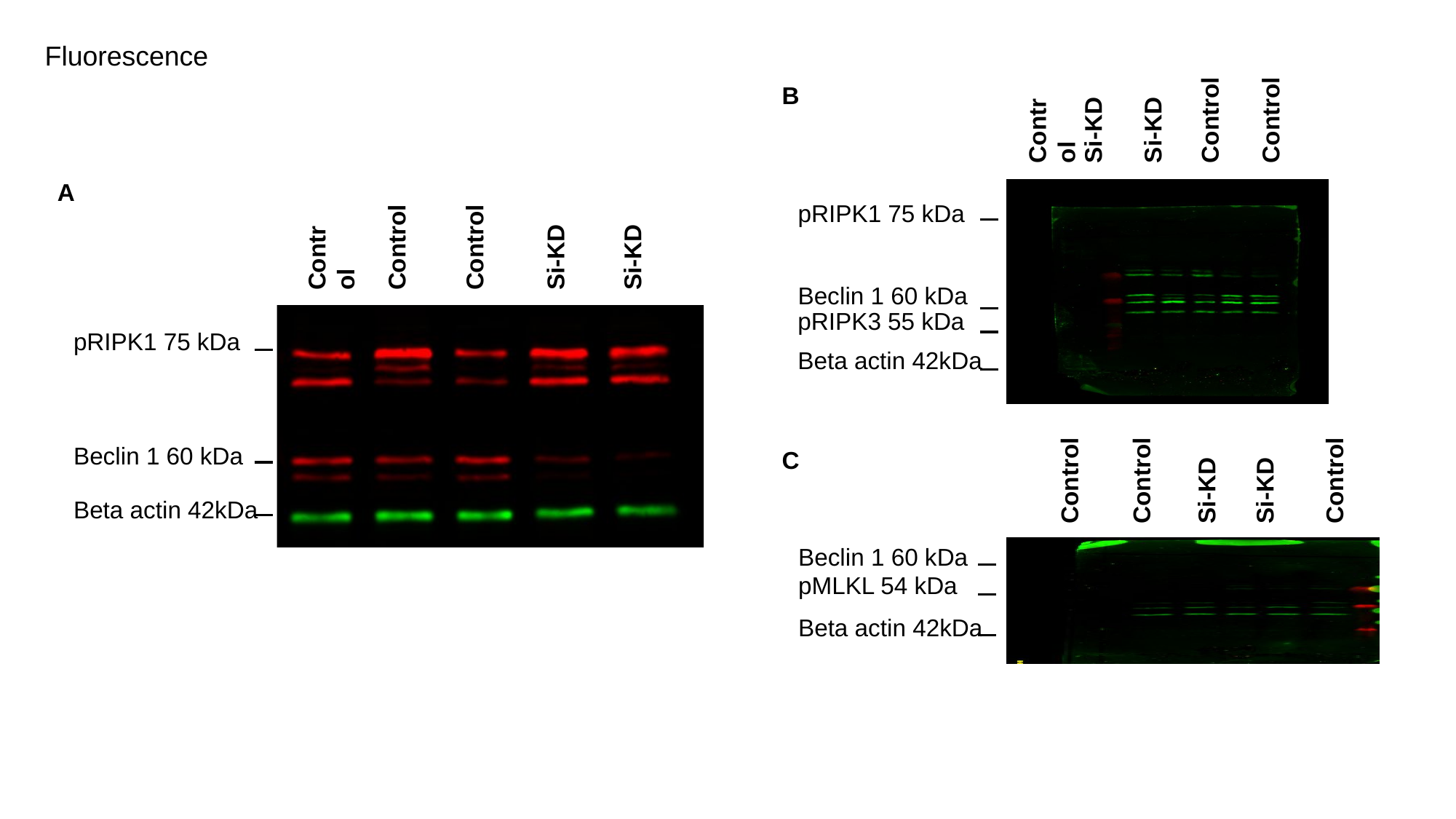

Fluorescence
Si-KD
Si-KD
Control
Control
B
Control
pRIPK1 75 kDa
A
Beclin 1 60 kDa
Control
Control
Control
Si-KD
Si-KD
pRIPK3 55 kDa
pRIPK1 75 kDa
Beta actin 42kDa
Beclin 1 60 kDa
Si-KD
Si-KD
Control
Control
Beta actin 42kDa
Control
C
Beclin 1 60 kDa
pMLKL 54 kDa
Beta actin 42kDa

## Slide 5
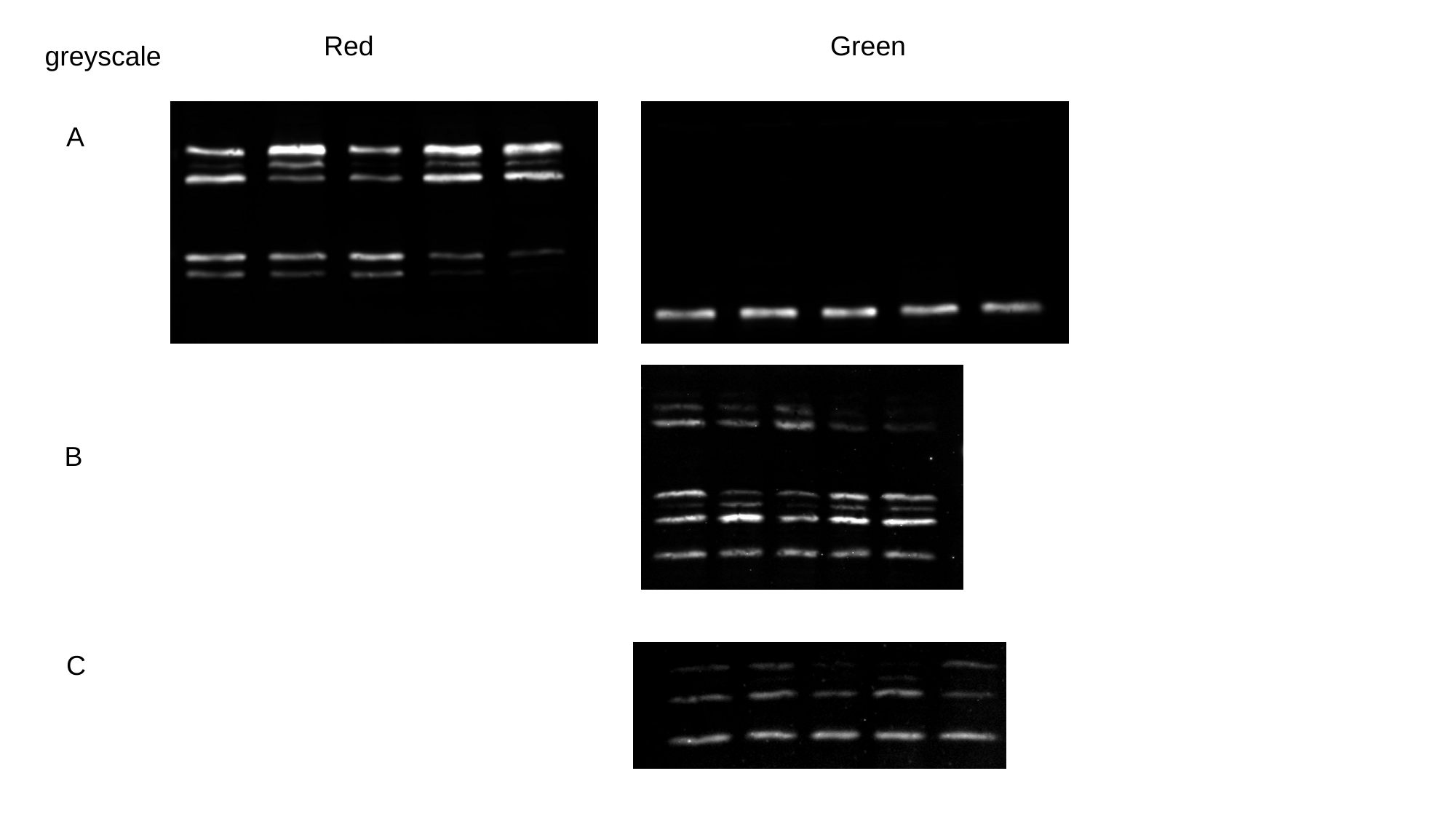

Red
Green
greyscale
A
B
C

## Slide 6
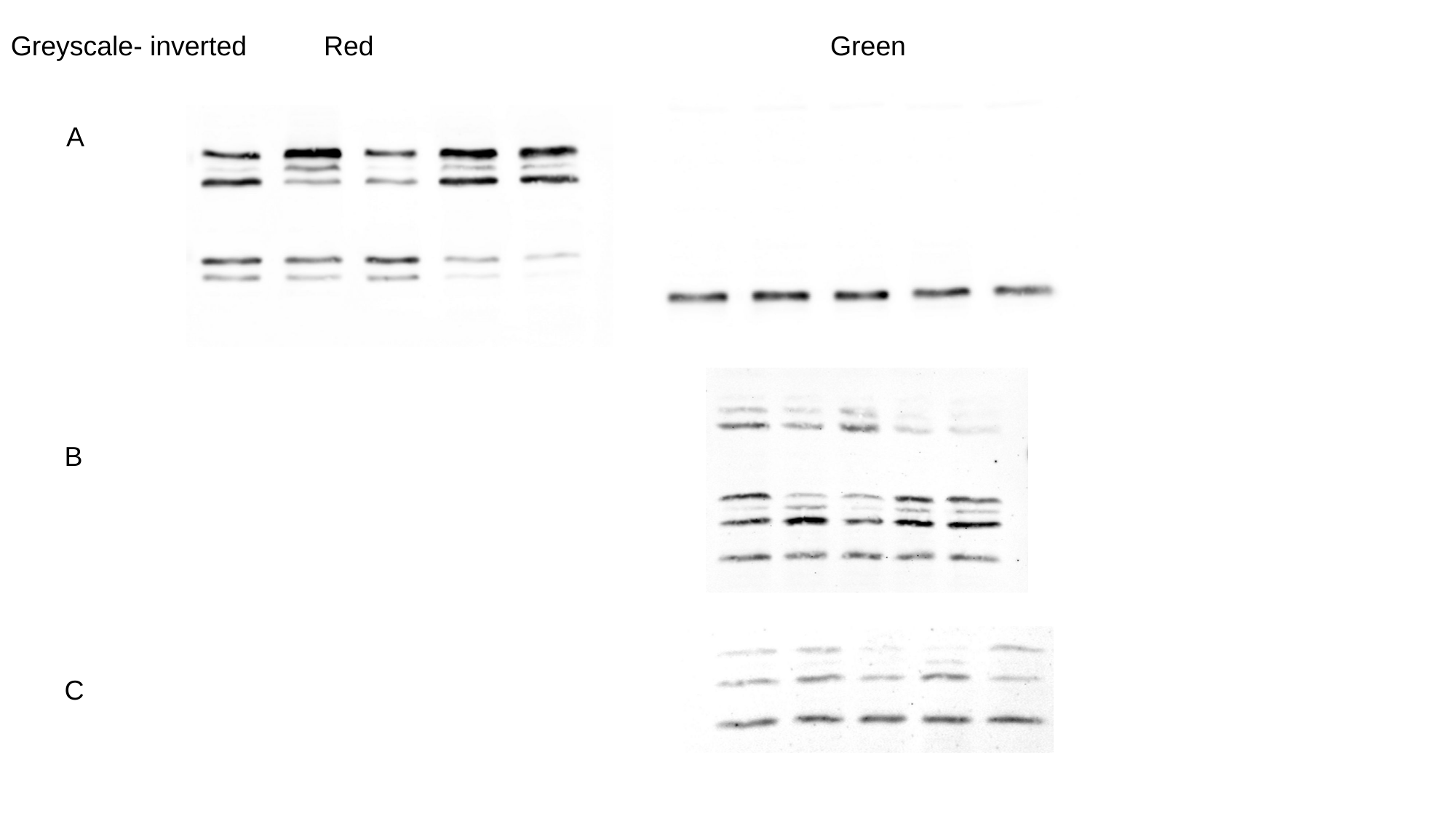

Greyscale- inverted
Red
Green
A
B
C

## Slide 7
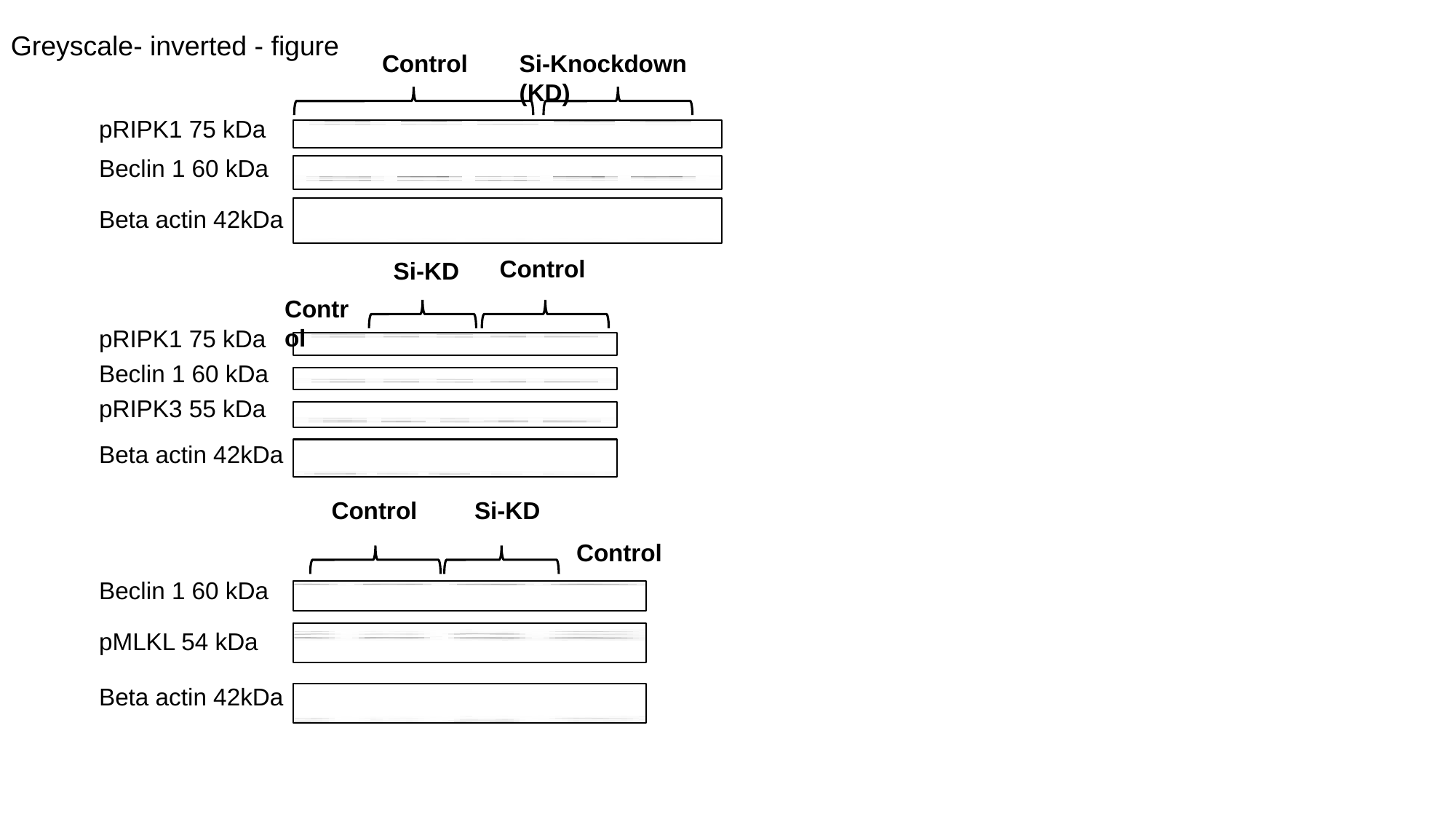

Si-Knockdown (KD)
Control
pRIPK1 75 kDa
Greyscale- inverted - figure
Beclin 1 60 kDa
Beta actin 42kDa
Control
Si-KD
pRIPK1 75 kDa
Control
Beclin 1 60 kDa
pRIPK3 55 kDa
Beta actin 42kDa
Control
Si-KD
Beclin 1 60 kDa
Control
pMLKL 54 kDa
Beta actin 42kDa

## Slide 8
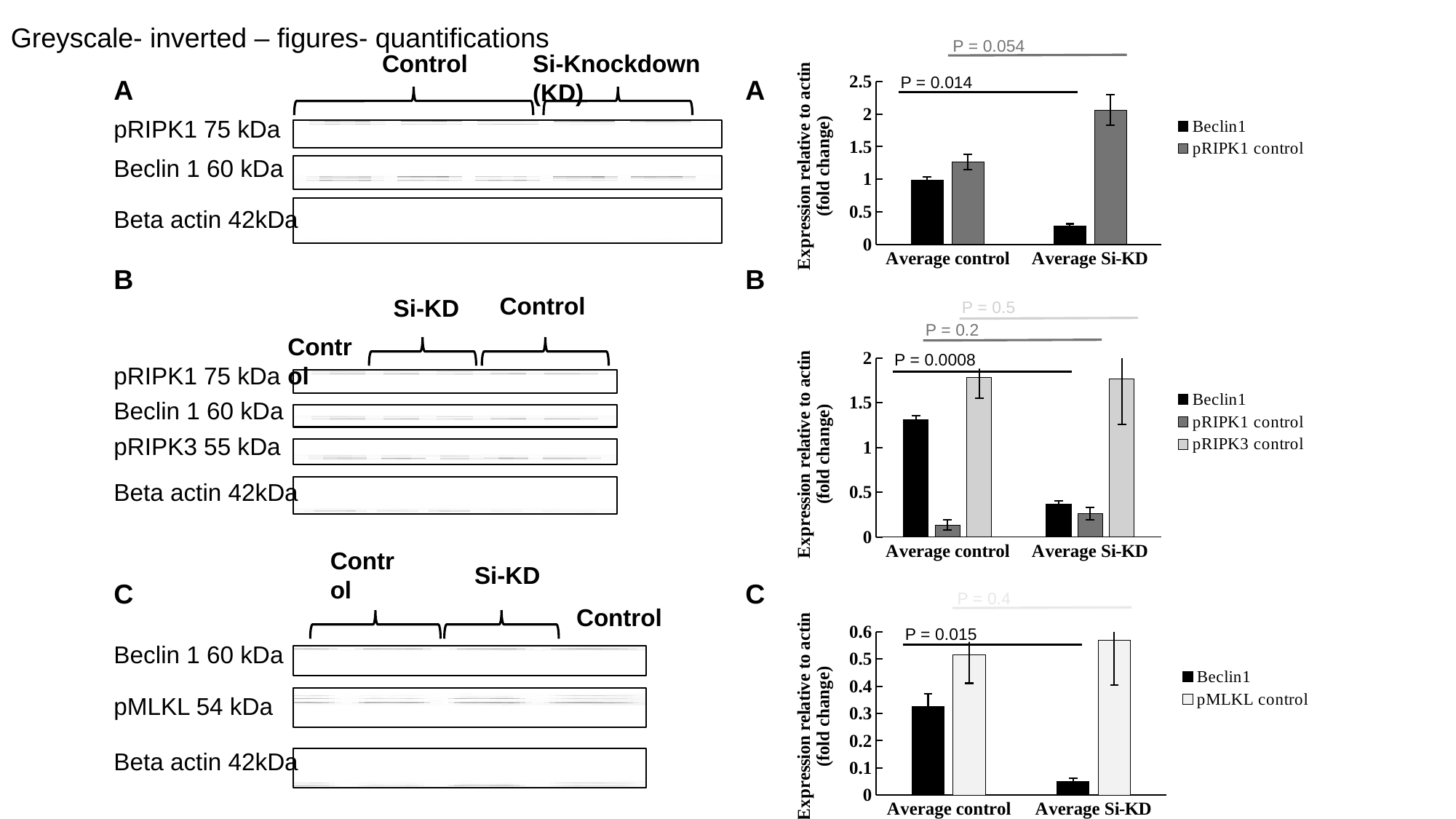

### Chart:
| Category | Beclin1 | pRIPK1 control |
|---|---|---|
| Average control | 0.9806391364554737 | 1.2631739218003255 |
| Average Si-KD | 0.28338593043103416 | 2.0609102549084426 |P = 0.054
P = 0.014
Greyscale- inverted – figures- quantifications
Si-Knockdown (KD)
Control
pRIPK1 75 kDa
Beclin 1 60 kDa
A
Beta actin 42kDa
A
Control
B
Si-KD
pRIPK1 75 kDa
Control
Beclin 1 60 kDa
pRIPK3 55 kDa
Beta actin 42kDa
B
### Chart:
| Category | Beclin1 | pRIPK1 control | pRIPK3 control |
|---|---|---|---|
| Average control | 1.3136134868822378 | 0.1339972656865274 | 1.779096933454407 |
| Average Si-KD | 0.36784064889398216 | 0.2576067593324884 | 1.767966790320732 |P = 0.5
P = 0.2
P = 0.0008
Control
Si-KD
Beclin 1 60 kDa
Control
C
C
pMLKL 54 kDa
Beta actin 42kDa
### Chart:
| Category | Beclin1 | pMLKL control |
|---|---|---|
| Average control | 0.326583726816662 | 0.515300092381071 |
| Average Si-KD | 0.04971976109504216 | 0.5696808277788015 |P = 0.4
P = 0.015

## Slide 9
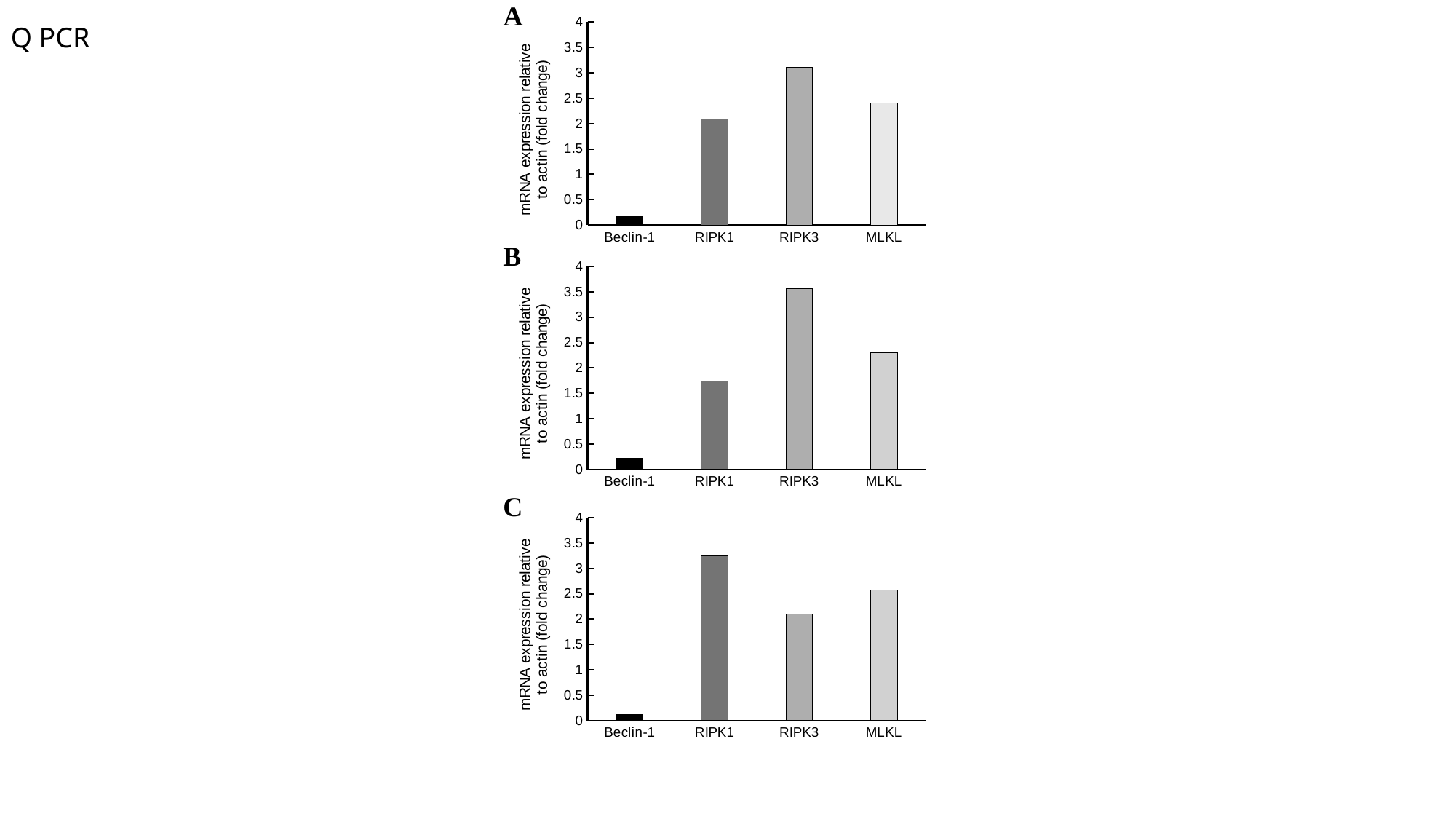

A
### Chart
| Category | Average fold-change |
|---|---|
| Beclin-1 | 0.16493848884661244 |
| RIPK1 | 2.0945882456412632 |
| RIPK3 | 3.1022895236674755 |
| MLKL | 2.4060500721642373 |Q PCR
B
### Chart
| Category | Average fold-change |
|---|---|
| Beclin-1 | 0.2227246795350852 |
| RIPK1 | 1.7411011265922491 |
| RIPK3 | 3.5635948725613456 |
| MLKL | 2.297396709994063 |C
### Chart
| Category | Average fold-change |
|---|---|
| Beclin-1 | 0.11935520048880237 |
| RIPK1 | 3.249009585424956 |
| RIPK3 | 2.0945882456412583 |
| MLKL | 2.5787406168791653 |

## Slide 10
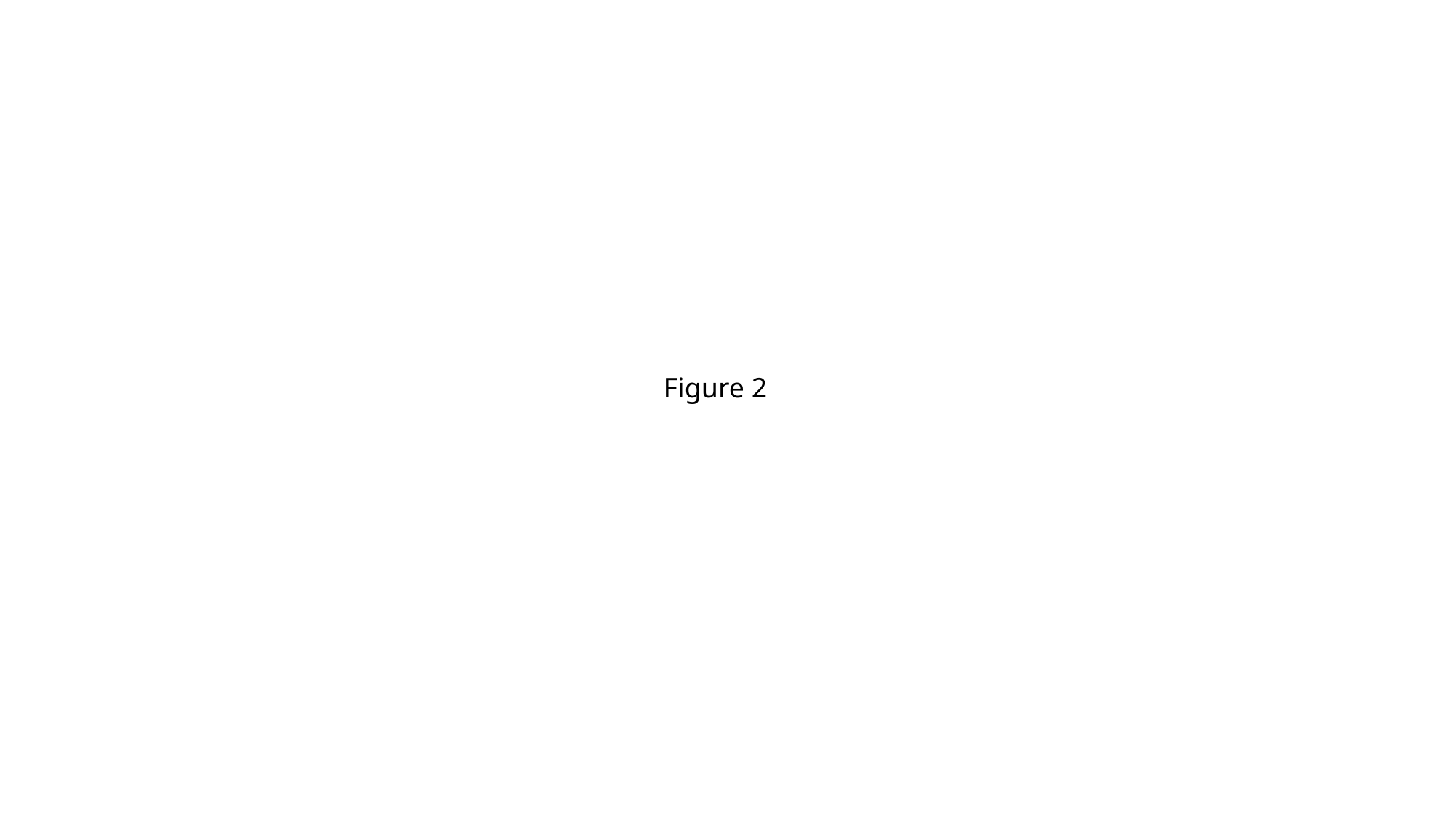

Figure 2

## Slide 11
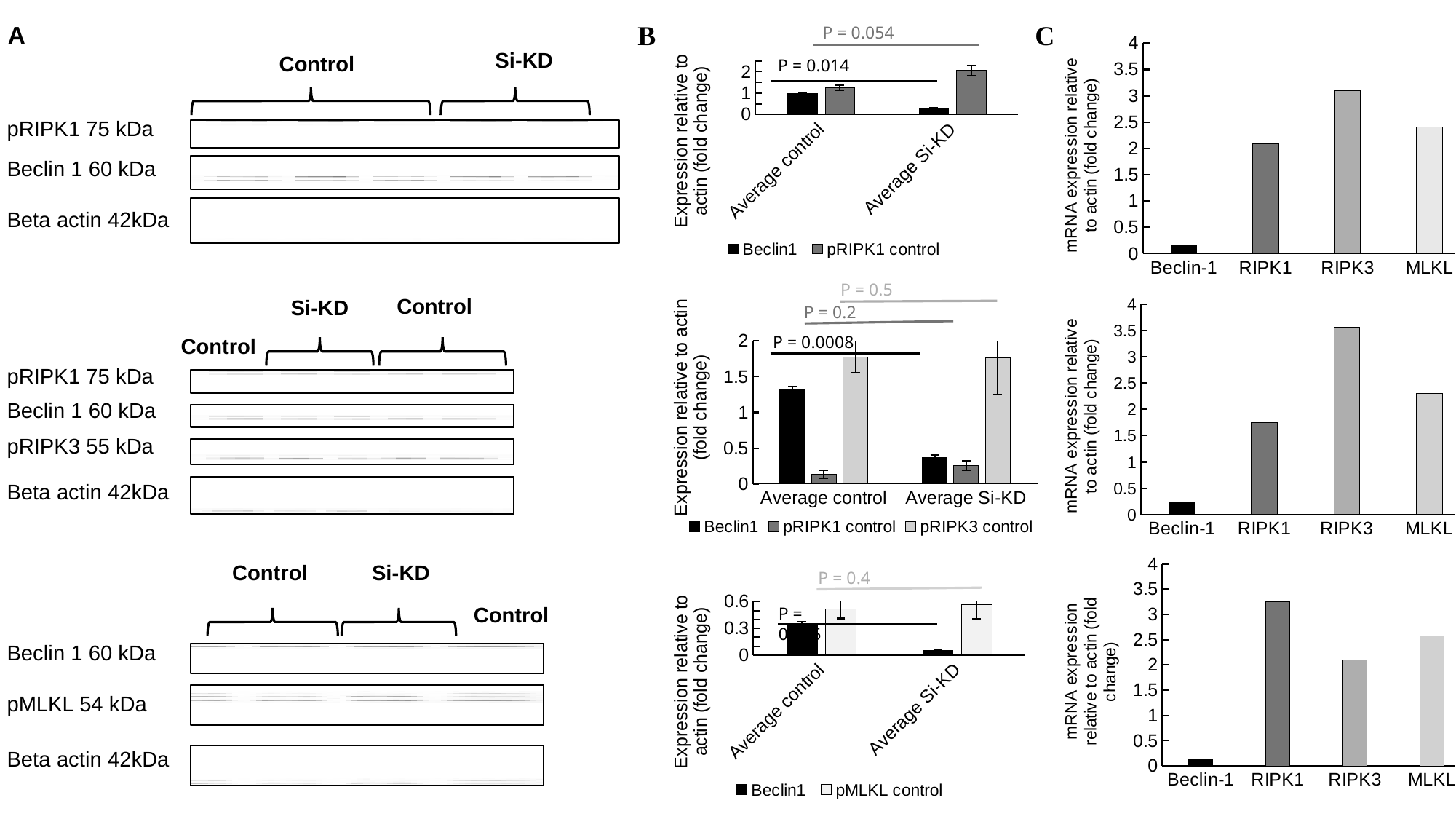

### Chart:
| Category | Beclin1 | pRIPK1 control |
|---|---|---|
| Average control | 0.9806391364554737 | 1.2631739218003255 |
| Average Si-KD | 0.28338593043103416 | 2.0609102549084426 |P = 0.054
P = 0.014
B
### Chart:
| Category | Beclin1 | pRIPK1 control | pRIPK3 control |
|---|---|---|---|
| Average control | 1.3136134868822378 | 0.1339972656865274 | 1.779096933454407 |
| Average Si-KD | 0.36784064889398216 | 0.2576067593324884 | 1.767966790320732 |P = 0.5
P = 0.2
P = 0.0008
### Chart:
| Category | Beclin1 | pMLKL control |
|---|---|---|
| Average control | 0.326583726816662 | 0.515300092381071 |
| Average Si-KD | 0.04971976109504216 | 0.5696808277788015 |P = 0.4
P = 0.015
A
Control
pRIPK1 75 kDa
Si-KD
Beclin 1 60 kDa
Beta actin 42kDa
Control
Si-KD
pRIPK1 75 kDa
Control
Beclin 1 60 kDa
pRIPK3 55 kDa
Beta actin 42kDa
Control
Si-KD
Beclin 1 60 kDa
Control
pMLKL 54 kDa
Beta actin 42kDa
C
### Chart
| Category | Average fold-change |
|---|---|
| Beclin-1 | 0.16493848884661244 |
| RIPK1 | 2.0945882456412632 |
| RIPK3 | 3.1022895236674755 |
| MLKL | 2.4060500721642373 |
### Chart
| Category | Average fold-change |
|---|---|
| Beclin-1 | 0.2227246795350852 |
| RIPK1 | 1.7411011265922491 |
| RIPK3 | 3.5635948725613456 |
| MLKL | 2.297396709994063 |
### Chart
| Category | Average fold-change |
|---|---|
| Beclin-1 | 0.11935520048880237 |
| RIPK1 | 3.249009585424956 |
| RIPK3 | 2.0945882456412583 |
| MLKL | 2.5787406168791653 |
